# High Density Lipoprotein Stimulated Migration of Macrophages Depends on the Scavenger Receptor Class B, Type I, PDZK1 and Akt1 and Is Blocked by Sphingosine 1 Phosphate Receptor Antagonists

**DOI:** 10.1371/journal.pone.0106487

**Published:** 2014-09-04

**Authors:** Aishah Al-Jarallah, Xing Chen, Leticia González, Bernardo L. Trigatti

**Affiliations:** Department of Biochemistry and Biomedical Sciences, and the Thrombosis and Atherosclerosis Research Institute, McMaster University, Hamilton, Ontario, Canada; Harvard Medical School, United States of America

## Abstract

HDL carries biologically active lipids such as sphingosine-1-phosphate (S1P) and stimulates a variety of cell signaling pathways in diverse cell types, which may contribute to its ability to protect against atherosclerosis. HDL and sphingosine-1-phosphate receptor agonists, FTY720 and SEW2871 triggered macrophage migration. HDL-, but not FTY720-stimulated migration was inhibited by an antibody against the HDL receptor, SR-BI, and an inhibitor of SR-BI mediated lipid transfer. HDL and FTY720-stimulated migration was also inhibited in macrophages lacking either SR-BI or PDZK1, an adaptor protein that binds to SR-BI's C-terminal cytoplasmic tail. Migration in response to HDL and S1P receptor agonists was inhibited by treatment of macrophages with sphingosine-1-phosphate receptor type 1 (S1PR1) antagonists and by pertussis toxin. S1PR1 activates signaling pathways including PI3K-Akt, PKC, p38 MAPK, ERK1/2 and Rho kinases. Using selective inhibitors or macrophages from gene targeted mice, we demonstrated the involvement of each of these pathways in HDL-dependent macrophage migration. These data suggest that HDL stimulates the migration of macrophages in a manner that requires the activities of the HDL receptor SR-BI as well as S1PR1 activity.

## Introduction

Macrophages are phagocytic cells that play a key role in innate host defense against invading pathogens, environmental agents and in clearance of modified/damaged host cells/molecules [1]. Macrophages also play a key role in the development of atherosclerotic vascular disease. Atherosclerosis is characterized by the accumulation of cholesterol-engorged macrophages within the walls of arteries. These so called foam cells appear to be the most abundant cells within atherosclerotic plaques. Atherosclerosis is triggered by the retention of low density lipoprotein (LDL) in the walls of arteries, subsequent modification of LDL, for example by oxidation, and engulfment of modified LDL by macrophages [2]–[4]. Macrophage endocytosis of modified LDL is mediated by scavenger receptors such as the class A types I and II and CD36 proteins, in a manner that is not regulated by the accumulation of cellular cholesterol [1], [3]. This leads to the accumulation of large amounts of intracellular cholesterol stored in cytoplasmic cholesteryl ester droplets, giving the cells a characteristic foamy appearance.

A characteristic feature of macrophages in atherosclerotic plaques is their relative inability to migrate. Recently this has been linked to increased cellular cholesterol in macrophage-derived foam cells in atherosclerotic plaques as a consequence of endocytosis of modified lipoproteins [5]–[7]. In addition, hypoxia has also been implicated in reduced macrophage migration [8]. Migration of macrophages and dendritic cells, related phagocytic antigen presenting cells, has recently been shown to be critical for their egress out of plaques, a key step in the regression of atherosclerotic plaques [9]–[14]. Atherosclerotic plaque regression, or the reversal of pre-established atherosclerotic plaques, is an important goal in the design of anti-atherosclerosis therapies which would be administered to patients with pre-established disease [15]. Thus mechanisms for inducing macrophage migration in response to appropriate chemotactic factors which could lead to their egress out of atherosclerotic plaques are important for designing novel therapeutics aimed at stimulating atherosclerotic plaque regression. Animal models and human studies have both demonstrated that atherosclerotic plaque regression can be achieved by decreasing the concentration of circulating LDL and increasing the concentration of circulating high density lipoproteins (HDL) [11], [13], [16]–[19].

An inverse relationship between circulating levels of HDL and coronary heart disease has been reported in numerous clinical and epidemiological studies [20]–[22]. HDL particles as well as HDL associated proteins and lipids were shown to exert a broad scope of potentially anti-atherogenic effects [23]–[25]. These include the ability to mediate reverse cholesterol transport from atherosclerotic plaque resident foam cells to the liver [26]–[28]. HDL also exhibits various anti-inflammatory and anti-oxidative properties [23]–[25]. Short term weekly infusions of reconstituted HDL particles resulted in rapid and significant regression of coronary atherosclerosis in patients with acute coronary syndrome [16]. Similar, though more striking, results have been obtained in apolipoprotein (apo) E knockout (KO) mice injected with reconstituted HDL [29], [30]. A study of atherosclerotic plaque regression in mice has reported significant alterations in the expression of a variety of genes in inflammatory cells in the regressing plaques, including significantly increased expression of the scavenger receptor class B, type I (SR-BI) [13].

SR-BI is a high affinity HDL receptor that mediates selective HDL lipid uptake [31]. Data from genetically altered mice demonstrates that overexpression of SR-BI in livers protects against atherosclerosis while knockout of SR-BI either in all tissues or in bone marrow derived cells promotes atherosclerosis [32]–[34]. The interaction of HDL with SR-BI leads to both bi-directional lipid exchange between the bound particle and cells [31], [35] as well as the activation of various signaling pathways [23], [25], [36]. HDL induced activation of protein kinase C (PKC) was reported in Chinese hamster ovary-derived cells overexpressing SR-BI, and PKC activity, in turn, appears to increase the selective lipid uptake activity of SR-BI [37]-[39]. HDL dependent signaling mediated by SR-BI is also well demonstrated in endothelial cells, where the interaction of HDL with SR-BI activated various signaling pathways such as the PI3K-Akt, p38 MAPK and ERK1/2 resulting in induction of endothelial nitric oxide synthase (eNOS) and cell migration but suppression of adhesion molecules expression and apoptosis [25], [40]. Studies in endothelial cells demonstrated that cholesterol efflux, cholesterol binding to the C-terminal transmembrane domain of SR-BI and the adaptor protein, PDZK1, were all required for HDL-induced signaling [25], [41] (reviewed in[42], [43]). In contrast little is known about HDL-SR-BI induced signaling in macrophages.

PDZK1 (Postsynaptic Density Protein (PSD-95)/Drosophila Discs-Large (Dlg)/Tight-Junction Protein (ZO1)) is a 519 amino acid, 63 kDa adapter protein that contains four PDZ protein-protein interaction domains [44]–[46]. The first and third PDZ domain of PDZK1 interact with the last three amino acids, AKL, of SR-BI's C-terminal cytoplasmic tail [45], [47], [48]. Knockdown of PDZK1 or deletion of the last three amino acids of SR-BI's C-terminal tail has been shown to impair HDL-dependent signaling in endothelial cells [40], [41].

Some of the atheroprotective actions of HDL may involve the delivery of bioactive lipids to cells [49]–[51]. For example, sphingosine-1-phosphate (S1P) and related lysosphingolipids carried by HDL exert atheroprotective effects on the endothelium [51]–[55]. Reconstituted HDL containing apo A1, phosphatidyl choline and S1P can efficiently deliver S1P to S1P receptors on cells [56], [57]. S1P signaling is mediated by a family of five G-protein coupled receptors (GPCR's), the S1P receptors (S1PR's) 1–5, encoded by distinct genes [58], [59]. In macrophages, S1P suppresses proinflammatory cytokine production in response to lipopolysaccharide, apparently through S1PR1 [60]. In endothelial cells, S1P increases eNOS activity and abundance and induces cell migration and survival via S1PR1 and S1PR3 [61] while HDL associated S1P inhibits the expression of adhesion molecules via S1PR1 and partially via S1PR3 [40] and improves endothelial barrier function via S1PR1 [62]. S1P induced endothelial cell migration involves Rho kinase [61], [63], p38 MAPK [63], [64] and PI3-Akt-Rac [65]–[67] pathways. S1P can be supplied exogenously to cells or generated intracellularly [58], [59]. S1P in the circulation is carried primarily by HDL [51]–[53], [55]. This, coupled with SR-BI's ability to mediate selective lipid uptake from HDL into cells, suggests that SR-BI might mediate HDL signaling in part by mediating the transfer of S1P from HDL into cells.

Given the ability of HDL to stimulate the migration of endothelial cells and the role of SR-BI and S1P receptors in that process, and given the importance of macrophage migration for atherosclerotic plaque regression, we hypothesized that HDL might also directly stimulate the migration of macrophages and that this might be mediated by SR-BI and S1PR's. We demonstrate that HDL can directly induce the migration of macrophages in a manner dependent on both SR-BI and on PDZK1. HDL stimulated migration also depends on S1PR1 activity, and is sensitive to the inactivation of PI3K-Akt1, p38 MAPK, ERK1/2, PKC and Rho kinase signaling pathways, known to be downstream of S1PR1. These data suggest that HDL stimulated macrophage migration may be one pathway by which HDL protects against atherosclerosis development and promotes plaque regression, and that this process is mediated by a signaling pathway involving SR-BI, PDZK1, S1PR1 and Akt1.

## Materials and Methods

### Ethics statement

All procedures involving mice were approved by the McMaster University Animal Research Ethics Board and were in accordance with the guidelines of the Canadian Council on Animal Care. All mice used were euthanized humanely under general anesthesia.

### Materials

All materials for cell culture, alexafluor-488-phalloidin and SYBR green were from Invitrogen/Life Technologies Inc. (Burlington ON, Canada). HDL, acetylated LDL (AcLDL) and apoA1 were from Biomedical Technologies, Inc (Stoughton MA, USA). Corning Transwell inserts, cell culture plastic-ware and Camco Quik Stain II were from VWR International (Mississauga, ON Canada). W146 and VPC23019 were from Avanti Polar Lipids (Alabaster AL, USA). Wortmannin, LY294002, SB203580, PD98059, Go6976 and Ro31-8220 were from EMD Millipore Corp. (Billerica, MA, USA). FTY720, SEW2871 and Y-27632 were from Cedarlane Labs (Burlington, ON Canada). Rat tail collagen I and FITC-labeled anti-rabbit IgG were from BD Biosciences (Mississauga, ON, Canada). Reagents for RNA isolation and cDNA synthesis were from Qiagen Inc. (Toronto, ON, Canada). All other suppliers were as indicated. All other reagents, including primers for qRT-PCR were from Sigma-Aldrich Canada Co. (Oakville, ON, Canada).

### Mice

All mice were on a C57BL6 background, except PDZK1 KO mice, which were on a mixed C57BL/6J X 129S6 background. C57BL6 mice were bred from founders purchased from the Jackson Laboratories (Bar Harbor ME, USA). SR-BI KO mice, originally provided by Monty Krieger (Massachusetts Institute of Technology), were backcrossed 10 times onto a C57BL/6J background before interbreeding. Akt1, 2 and 3 KO mice were bred from founders provided by Morris Birnbaum (University of Pennsylvania). PDZK1 KO mice and corresponding WT mice were from Jackson Laboratories. All mice were bred and housed in the Thrombosis and Atherosclerosis Research Institute Division of Comparative Medicine and provided with free access to food and water.

### Cells and cell culture

Cells were cultured at 37°C in an atmosphere of 5% CO_2_in air. RAW 264.7 cells were cultured in DMEM supplemented with 10% heat-inactivated FBS, 2mM L-glutamine and 50 µg/ml penicillin/streptomycin and were passaged by scraping. Primary mouse peritoneal macrophages (MPM's) were isolated from mice injected intraperitoneally on day 0 with 1ml of 10% thioglycolate. On day 4 the mice were anesthetized with isoflurane gas and euthanized humanely by cervical dislocation and peritoneal cells were collected with 10 ml PBS containing 5mM EDTA. The cells were washed once in DMEM containing 20% FBS and plated in DMEM containing 10% FBS (8×10^6^cells/10 cm dish). Foam cells were prepared by incubating MPM's with AcLDL (100 µg protein/ml) for 48 hrs. Prior to migration experiments, cells were washed twice with serum fee media and cultured for 16 hrs either in the absence of serum or in the presence of 3% newborn calf lipoprotein deficient serum (NCLPDS) [68] as indicated.

### Macrophage migration and adhesion assays

Macrophage migration was tested in a chemotaxis assay [5], [7] using Transwell inserts with pore size of 5 µm that were pre-coated with rat tail collagen I (110 µg/ml). Cells (4×10^5^) were added to the upper chamber and incubated for 2 hrs at 37°C in media containing 5% NCLPDS. The media was then removed and replaced with media containing 0.5% NCLPDS. These filter inserts were placed in wells containing the same media with either no further additives or with one of the following: HDL or apoA1 (100 µg protein/ml) [69], FTY720 (2 ng/ml) [70] or monocyte chemotactic protein-1 (MCP-1; 100 ng/ml [71], Fitzgerald Industries International, Acton, MA USA). In migration assays testing the effects of immunological or pharmacological inhibitors, the cells were pre-incubated with the inhibitor for 30 min and the migration assays were performed in the continued absence or presence of the inhibitors. The following compounds were used at concentrations previously reported to be effective: anti-SR-BI blocking antibody (KKB-1, 0.5 µg/ml [72], generously provided by Karen Kozarsky, ReGenX Biosciences), BLT-1 (0.3 µM [37], [73], ID 5234221, ChemBridge Corp.), VPC23019 (10 µM) [74], pertussis toxin (PTX, 100 ng/ml) [75], LY294002 (10 µM) [76], SB203580 (1 µM) [77], PD98059 (10 µM) [78], Go6976 and Ro31-8220 (5 µM) [79], W-146 (10 µM) [80], SEW2871 (5nM) [81] and Y-27632 (10 µM) [82]. The following reagents were added as 1000× stock solutions in DMSO: BLT-1, VPC23091, LY294002, SB203850, PD98059, Go6976, Ro31-8220, W-146, and Y-27632. PTX was prepared as a 100 µg/ml stock in sterile water containing 2 mg/ml BSA. SEW2871 was prepared as a 100 mM stock in dimethylformamide. For each experiment, all samples were treated with an equivalent final concentration of solvent vehicle for each reagent used. After 4 hrs, cells on the filters were fixed and stained with CamcoQuick Stain II or with 300 nM 4′,6-diamidino-2-phenylindole dihydrochloride (DAPI) for 10 min and rinsed twice with water. The non-migrated cells on the upper surface of the filters were removed by carefully scraping with a cotton swab and the cells that had migrated to the lower face of each filter were visualized by bright-field or fluorescent microscopy (using a Carl Zeiss Axiovert 200 M inverted microscope with a 10× objective) and counted. Cell adhesion to collagen I was tested by plating the same number of cells (as for migration assays) in each well of a 96 well dish pre-coated with rat tail collagen I under the same conditions as those used to coat the Transwell filters. After 4 hrs non-adhered cells were washed away and adherent cells were fixed, stained as above with DAPI, and cell adhesion was measured by counting DAPI stained nuclei.

### Phalloidin staining

Cells were cultured on sterile, untreated glass coverslips, as above, in the presence or absence of HDL (100 µg protein/ml) for different times. The cells were then washed twice with PBS, fixed with 3% paraformaldehyde pH 7.4 for 30 min and permeabilized with 0.1% Triton-X100 for 5 min. at room temperature. F-actin was stained using 25units/ml Alexafluor488 phalloidinin PBS for 30 min at room temperature. The coverslips were then mounted using Vectasheild (Vector Laboratories Canada Inc. Burlington, ON, Canada) and imaged using a Zeiss Axiovert 200 M fluorescence microscope with standard FITC filters using a 40× objective.

### SDS-PAGE and immunoblotting

RAW 264.7 cells were serum starved overnight and incubated with HDL (100 µg protein/ml) for different times, washed and lysed in ice cold lysis buffer containing: 0.2xPBS, 0.1% Triton-X100, 1× Phosphatase Inhibitor Cocktail 2 (Sigma Aldrich catalogue number P5726) and protease inhibitors (20 µg/ml aprotinin, 10 µg/ml leupeptin, 1 mM APMSF and 10 µg/ml pepstatinA). To prepare total membranes, MPMs were homogenized on ice for 1 min in 20 mM Tris-HCl, pH 7.5 containing 2 mM MgCl_2_, 0.25 M sucrose, and protease inhibitors with the concentrations indicated above. Homogenates were centrifuged at 3000xg for 10 min at 4°C and the supernatant was subjected to another centrifugation step at 100,000xg for 1 hr at 4°C. The pellet was then suspended in 10 mM sodium phosphate, pH 7.0 containing the protease inhibitors listed above. After boiling, the samples were subjected to SDS-PAGE followed by immunoblotting with rabbit anti-Akt, rabbit anti-phospho-Akt (Ser473) (Cell Signaling Technology, Danvers, MA, USA). HRP-conjugated donkey-anti-rabbit and donkey-anti-mouse antibodies (Jackson ImmunoResearch Labs Inc, West Grove PA, USA) were used as secondary antibodies and were detected using Western Lightning ECL reagent kit (PerkinElmer Canada, Woodbridge ON, Canada).

### Flow cytometry

SR-BI surface expression was measured in unfixed non-permeabilized MPMs. Macrophages were treated with rat anti-CD16/32 (eBioscienceInc, San Diego, CA, USA) to block FC receptors then incubated with rabbit anti-SR-BI antibody (KKB-1, 0.5µg/ml) followed by a FITC-conjugated anti-rabbit IgG. Cell sorting was performed using BD FACSCalibur instrument and data was analyzed using Cell Quest Pro software [73].

### Quantitative RT-PCR

Total RNA was extracted from lungs and peritoneal macrophages of wild type mice using RNeasy kit. cDNA was generated from 1 µg of total RNA using Quantitec Reverse Transcription kit. Real-time PCR was performed using the SYBR green detection method as described [83]. Primers were as follows[84]: Mouse S1PR1forward 5′-ACT TTG CGA GTG AGC TG-3′ reverse 5′-AGT GAGCCT TCA GTT ACA GC-3′; S1PR2 forward 5′-TTC TGG AGG GTA ACACAG TGG T-3′ reverse 5′-ACA CCC TTT GTA TCA AGT GGC A-3′; S1PR3 forward 5′-TGG TGT GCG GCT GTC TAG TCA A-3′ reverse 5′-CAC AGC AAG CAG ACC TCC AGA-3′; GAPDH forward 5′-ACCACAGTCCATGCCATCAC-3′ reverse 5′-TCCACCACCCTGTTGCTGTA-3′. Results were calculated as described [85].

### Statistical analysis

Data was analyzed with Sigma Plot Software using the Student's T-test for pairwise comparisons between 2 groups, or using one way ANOVA with Holm-Sidak post hoc test or two way ANOVA with Tukey's post hoc test for comparisons of multiple groups where appropriate and was considered statistically significant when P<0.05.

## Results

### HDL induces macrophage migration in SR-BI and PDZK1 dependent manner

**Figure 1:**
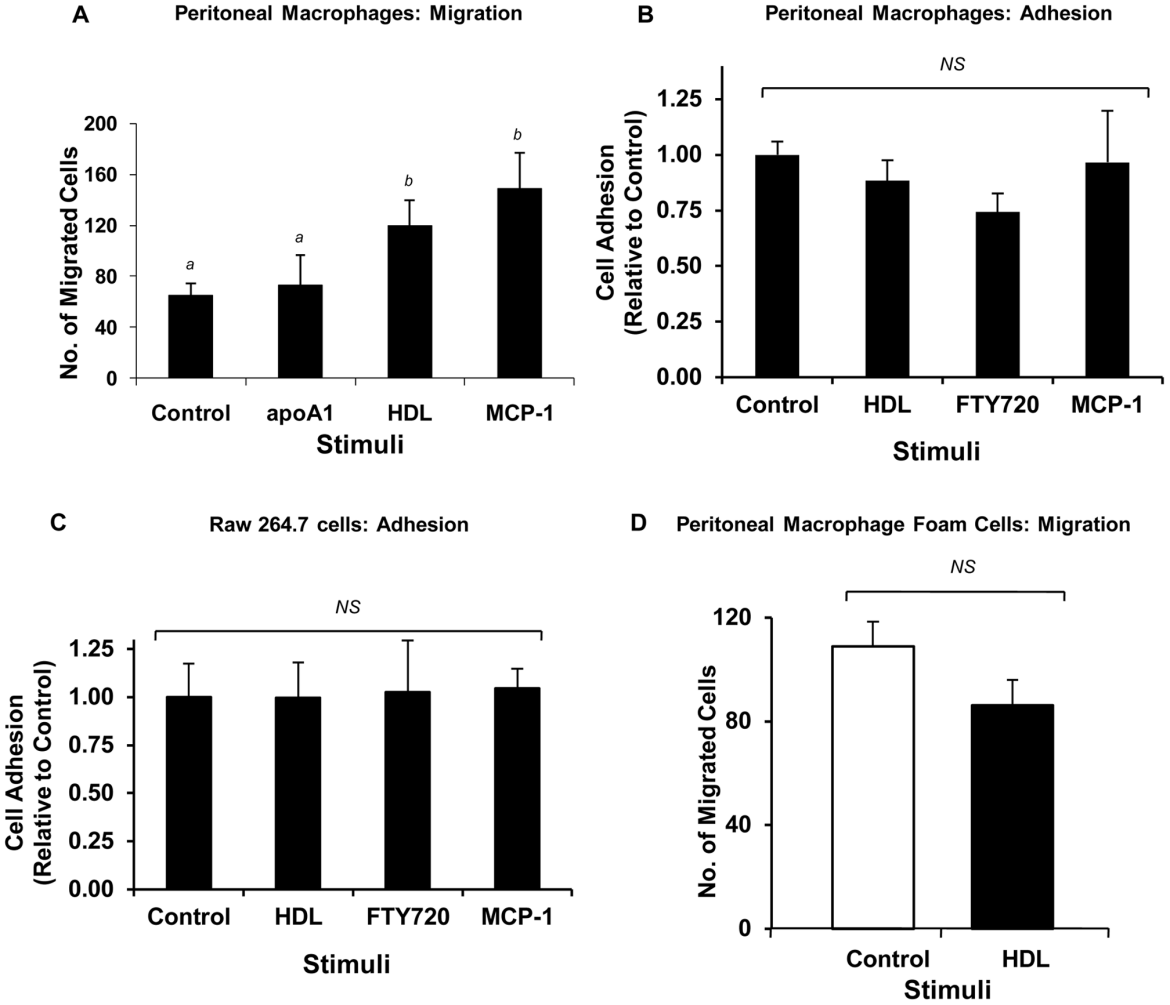
HDL stimulates the migration of macrophages but not of lipid loaded macrophage foam cells. **A.** Wild type mouse peritoneal macrophages were incubated in lipoprotein deficient serum overnight and cell migration in response to no stimulus (control), apoA1(100 µg/ml), HDL (100 µg protein/ml) or MCP-1 (100 ng/ml) (each added to the bottom well of the migration assay chamber) was performed as described in the Methods section. The number of migrated cells/well from three independent samples from each group is represented as the mean ± standard deviation. **B.** Wild type mouse peritoneal macrophages, or **C.** RAW 264.7 cells were incubated in collagen I coated cell culture dishes with either HDL (100 µg protein/ml), FTY720 (2 ng/ml) or MCP-1 (100 ng/ml) under conditions paralleling the migration assay. Cell adhesion was measured by counting DAPI stained nuclei. The degree of cell adhesion was normalized to that in control cells and is represented as the mean ± standard deviation of triplicates. **D.** Foam cells from wild type mouse peritoneal macrophages were generated by culture for 48 hrs in the presence of AcLDL (100 µg/ml). Cells were washed and migration in response to HDL was measured as described in the Methods section. The number of migrated cells/well is plotted as the mean ± standard deviation of triplicates. Statistical analysis was done using one way ANOVA with Holm-Sidak post hoc test (A–C) or Student's T-test (D). Values identified with different letters are statistically significantly different (P<0.05). NS indicates that no statistically significant difference was detected.

We used a standard three-dimensional Transwell migration assay to examine if HDL might stimulate macrophage migration. In this set up, cells were added to the upper compartment of a cell culture dish, separated from the lower compartment by a collagen I coated membrane with 5 µm diameter pores. After allowing the cells to adhere to the collagen I coated membrane for 2 hrs, HDL, lipid free apoA1, the major protein component of HDL, and MCP1 were added to the lower compartment of individual wells. We also included, as a control, wells in which no stimulus was added. After 4 hrs at 37 °C, inserts were removed and the numbers of macrophages that had migrated from the upper to the lower face of the porous membrane were quantified. MCP-1, a well-known chemotactic factor for macrophages, at 100 ng/ml, stimulated a greater than 2-fold increase in migration of wild type mouse peritoneal macrophages from the upper to the lower face of the porous membrane. HDL, but not lipid free apoA1, at 100 µg protein/ml, induced a similar increase in migration (Figure 1A). To ensure that increased macrophage migration stimulated by either MCP-1 or HDL was not merely the consequence of increased macrophage adhesion to collagen I, which coated the porous membrane of the migration chamber, we tested the effects of each of these on the adhesion of macrophages to collagen I coated wells of a 96 well microtitre dish. Neither HDL, MCP-1, nor the known chemotactic factor FTY720, affected adhesion of either primary mouse peritoneal macrophages (Figure 1B) or RAW 264.7 cells (Figure 1C) to collagen I. In contrast to freshly isolated macrophages, peritoneal macrophages that were lipid-loaded by culture for 48 hrs in the presence of AcLDL failed to migrate in response to HDL (Figure 1D). This is consistent with previously reported findings that cholesterol loading of macrophages reduced their ability to migrate [5], [7].

**Figure 2:**
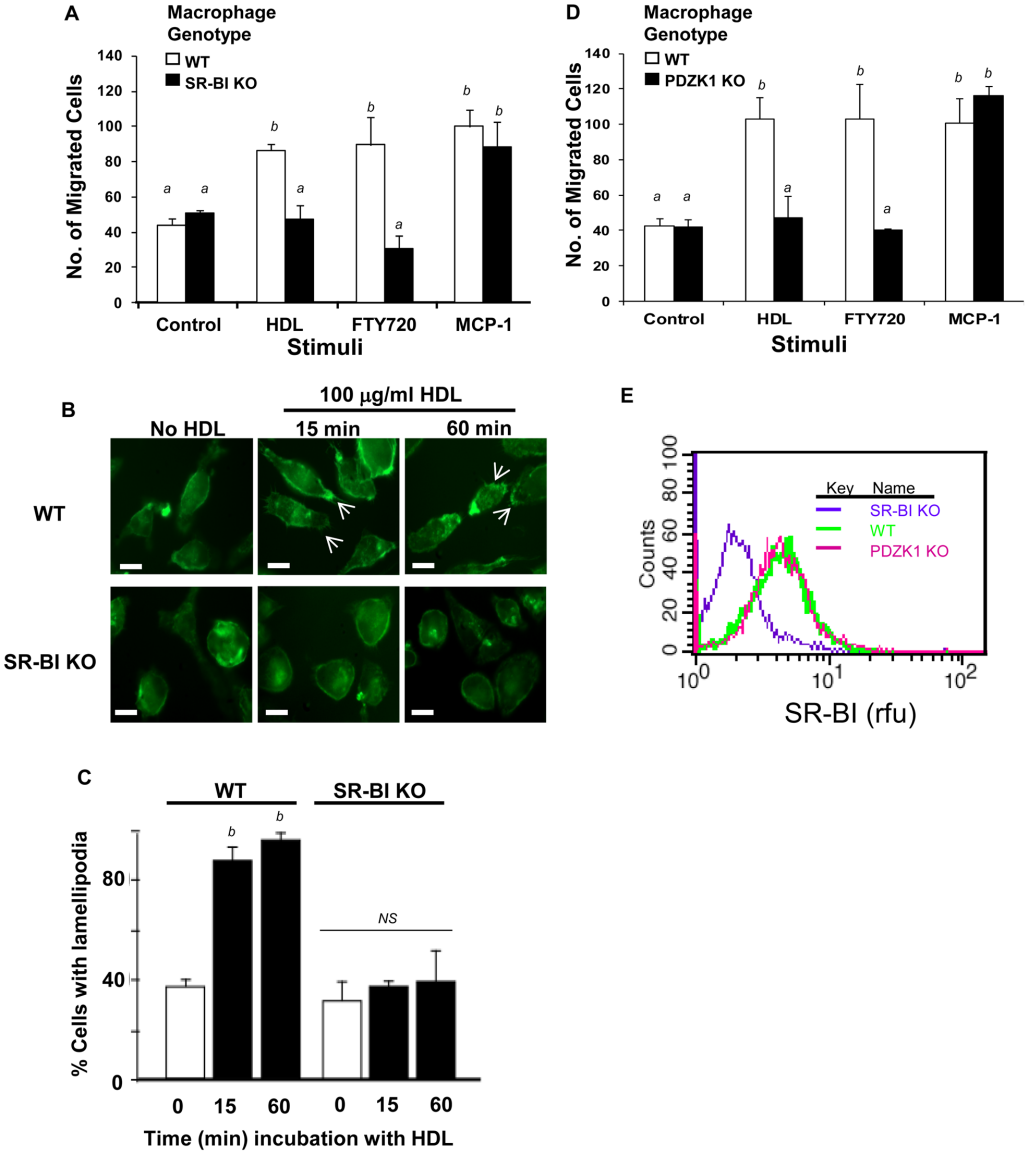
HDL-induced migration is reduced in macrophages deficient in either SR-BI or PDZK1. **A.** Peritoneal macrophages were prepared from either wild type (WT) or SR-BI KO mice, and cultured in the presence of 3% NCLPDS for 16 hrs before analysis. Migration of cells in response to no stimulus (control), HDL (100 µg protein/ml), FTY720 (2 ng/ml) or MCP-1 (100 ng/ml) as described in the legend to Figure 1 and the Methods section. **B.** Peritoneal macrophages from WT or SR-BI KO mice were cultured as described in A, prior to incubation with or without HDL (100 µg protein/ml) for the times indicated. Actin filaments were visualized by fluorescence microscopy after alexa 488-phalloidin staining. Representative images are shown. Scale bars = 10 µm. **C.** Numbers of cells with lamellipodia (arrows in B) were counted (∼100 cells over 4–5 fields) for cells isolated from three mice from each genotype. **D.** Migration of peritoneal macrophages from WT or PDZK1 KO mice as described for panel A. **E.** Flow cytometry analysis of cell surface SR-BI levels in wild type, SR-BI KO and PDZK1 KO macrophages. Values in A, C and D are means ± standard deviations of triplicates and are representative of multiple independent assays. Data in E is representative of multiple analyses. Statistical analysis in panels A, C and D was done using two way ANOVA with Tukey post hoc test. Values identified with different letters are statistically significantly different (P<0.001). NS indicates that no statistically significant difference was detected.

The HDL receptor SR-BI has been reported to mediate HDL signaling leading to migration of endothelial cells (25). To explore if SR-BI was required for macrophage migration in response to HDL we examined the ability of peritoneal macrophages prepared from SR-BI KO mice to migrate in response to HDL (Figure 2A). Unlike macrophages from wild type mice, SR-BI KO macrophages were not able to migrate in response to HDL. Similarly, they were also unable to migrate in response to the S1PR pro-agonist, FTY720, although they showed normal migration in response to MCP-1 (Figure 2A). Exposure of SR-BI expressing wild type mouse peritoneal macrophages to 100 µg/ml HDL resulted in a relatively rapid (within 15 min) rearrangement of the actin cytoskeleton and increased the formation of lamellipodia, as revealed by fluorescence microscopic analysis of fixed cells stained with alexa-488 labeled phalloidin (Figure 2B, upper panels, quantified in Figure 2C). Similar results were obtained using murine RAW 264.7 macrophage-like cells (not shown). These alterations in the actin cytoskeleton in response to HDL were not observed in peritoneal macrophages prepared from SR-BI deficient mice (Figure 2B, lower panels, quantified in Figure 2C). Peritoneal macrophages prepared from PDZK1 KO mice were also unable to migrate in response to HDL or to FTY720, although their ability to migrate in response to MCP-1 was unchanged (Figure 2D). Flow cytometry analysis confirmed that as previously reported by others, [86], knockout of PDZK1 did not alter the levels of SR-BI on the cell surface of macrophages (Figure 2E).

**Figure 3:**
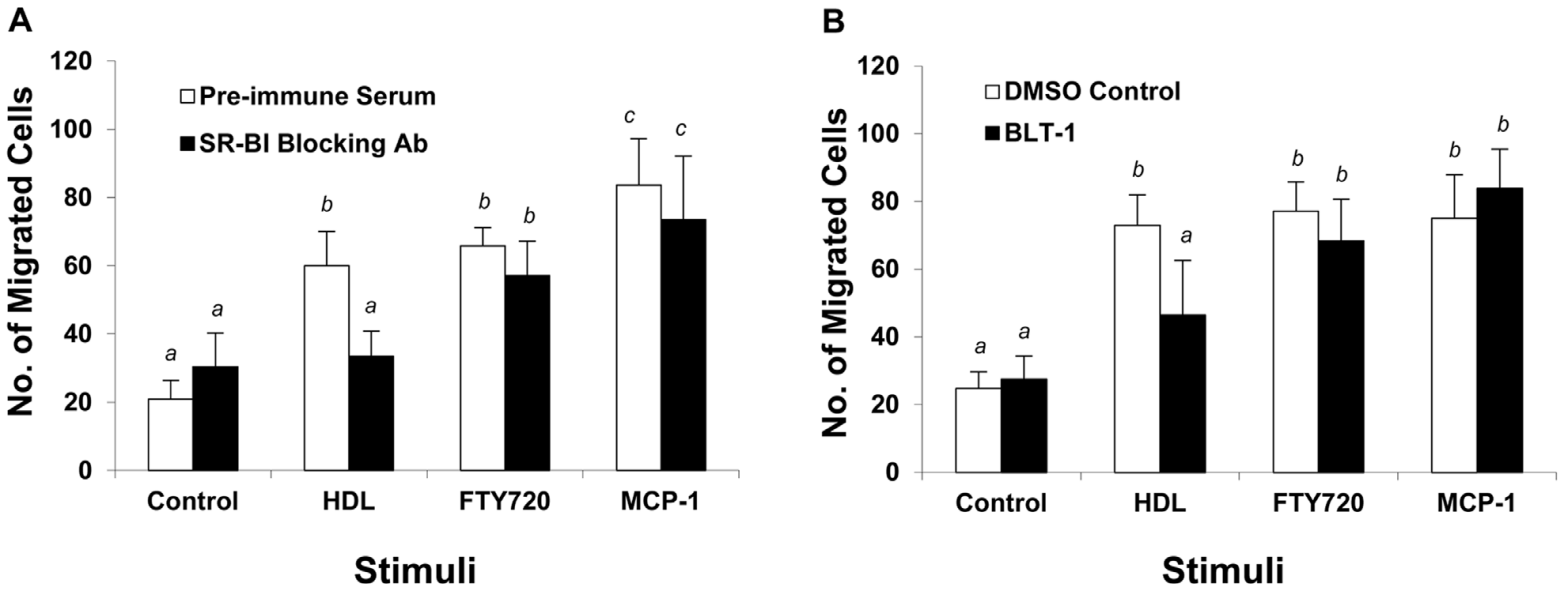
Antibody or small molecule mediated inactivation of SR-BI inhibits HDL dependent but not FTY720 dependent macrophage migration. Mouse peritoneal macrophages were pretreated for 30 min with either **A.** An anti-SR-BI blocking antiserum or pre-immune rabbit serum (0.5 µg/ml); or **B.** BLT-1 (0.3 µM), an inhibitor of SR-BI mediated lipid transfer, or DMSO vehicle control. Cell migration in response to HDL (100 µg protein/ml), FTY720 (2 ng/ml) or MCP-1 (100 ng/ml) was measured in the continued presence of the inhibitors as described in the legend to Figure 1 and the Methods section. Data are means ± standard deviations of 6 replicates. Statistical analysis was done using two way ANOVA with Tukey post hoc test. Values identified with different letters are statistically significantly different (P<0.002).

To determine if acute inactivation of SR-BI-mediated HDL binding or lipid transfer activity affected the ability of cells to migrate in response to HDL or FTY720, we tested the effects of pre-treating cells with either an anti-SR-BI blocking antibody, previously reported to prevent HDL binding to SR-BI, or with a small molecule, BLT-1 previously reported to selectively inhibit SR-BI mediated lipid transfer between bound HDL and cells without inhibiting HDL binding to SR-BI [73]. Immunological blockade of SR-BI substantially reduced HDL stimulated migration of peritoneal macrophages from wild type mice (Figure 3A). Treatment with BLT-1 also reduced HDL stimulated migration of peritoneal macrophages from wild type mice (Figure3B). This suggested that both HDL binding and lipid transfer activities of SR-BI are required for HDL stimulated migration of macrophages. Neither treatment affected the ability of macrophages to migrate in response to FTY720 or to MCP-1 or (Figure 3A, B). Therefore, acute inhibition of SR-BI selectively impairs migration in response to HDL; however unlike macrophages lacking SR-BI expression, those in which SR-BI activity is acutely blocked are still able to migrate in response to the S1PR pro-agonist, FTY720.

### HDL stimulated migration is dependent on a sphingosine-1-phosphate receptor

**Figure 4:**
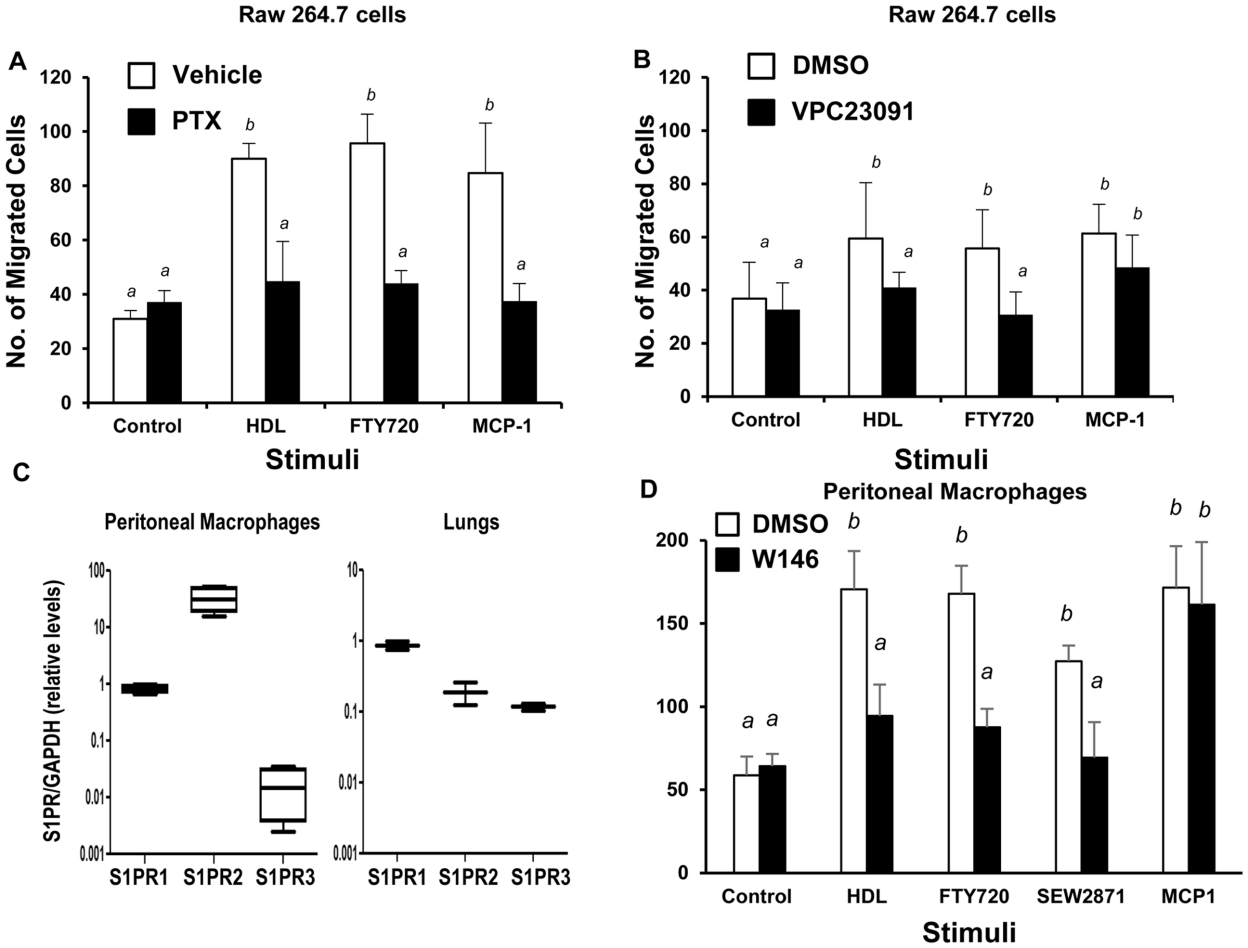
Blockade of S1PR1 prevents HDL stimulated macrophage migration. RAW 264.7 (panels A,B) or mouse peritoneal macrophages (panel D) were pretreated for 30 min with the following: **A.** PTX (100 ng/ml) an inhibitor of Gα_i_ protein coupled receptors; **B.** VPC23019 (10 µM) an antagonist of S1PR's 1, and 3; or **D.** W-146 (10 µM) an antagonist specific for S1PR1. Control cells were treated with either 2 µg/ml BSA or DMSO vehicle as indicated. Cell migration in response to HDL (100 µg protein/ml), FTY720 (2 ng/ml), MCP-1 (100 ng/ml) and/or SEW2871 (5 nM) was measured in the continued absence or presence of the inhibitors as described in the legend to Figure 1 and the Methods section. **C.** Analysis of expression of S1PR1, 2 and 3 in peritoneal macrophages (left panel) or lung tissue (right panel) from wild type mice. Data are means ± standard deviations of 6 replicates (A,B) or 3 replicates (C,D). Statistical analysis was done using two way ANOVA with Tukey post hoc test. Values identified with different letters are statistically significantly different (P<0.002).

FTY720 is a pro-agonist of S1PRs and S1P is a component of HDL. We therefore tested the effects of different inhibitors of S1PRs on macrophage migration stimulated by HDL, FTY720, and MCP-1. Like primary wild type mouse peritoneal macrophages (e.g. Figure 2 A), murine RAW 264.7 macrophage like cells were stimulated to migrate by HDL, FTY270 and MCP-1 (Figure 4 A,B). PTX, an inhibitor of the Gα_i_ subunit of heterotrimeric G proteins [87], inhibited the migration of RAW 264.7 cells in response to both FTY720 and, as expected, MCP-1 (Figure 4A), which acts through the chemokine receptor 2, known to be PTX-sensitive [88]. PTX treatment also inhibited migration in response to HDL (Figure 4A), indicating the involvement of a GPCR coupled to Gα_i_ in macrophage responses to HDL. Treatment of RAW 264.7 cells with VPC23019, an antagonist selective for S1PR1 and S1PR3 [89], blocked migration induced by FTY720 and HDL but not by MCP-1 (Figure 4B). We examined gene expression levels of three S1P receptors, S1PR1, 2 and 3 in mouse peritoneal macrophages and, for comparison, mouse lung tissue. Macrophages expressed S1PR1; S1PR2 levels were greater than 10-fold higher, whereas S1PR3 levels were approximately 100-fold lower than S1PR1 (Figure 4C). This is consistent with other reports of higher expression of S1PR1 than S1PR3 in macrophages [60], [90]. On the other hand, in lungs, S1PR1 levels appeared highest and S1PR2 and 3 levels were similar (at almost 10-fold lower than S1PR1). We focused our attention on S1PR1 as a potential mediator of HDL dependent stimulation of migration in macrophages because 1) HDL and FTY720 induced migration was blocked by PTX and VPC23019 which impair S1PR1 and 3 but not S1PR2 signaling [89], 2) S1PR1 expression in macrophages appeared to be much higher than S1PR3, and 3) S1PR2 has previously been reported to negatively regulate macrophage migration [91]. To confirm the involvement of S1PR1 in HDL stimulated macrophage migration, wild type mouse peritoneal macrophages were treated with the S1PR1-specific agonist, SEW2871, and antagonist W146 (Figure 4D). SEW2871, like HDL and FTY720, stimulated macrophage migration. Furthermore, HDL-, FTY720- and SEW2871- stimulated macrophage migration were blocked by treatment with the S1PR1 specific antagonist, W146 (Figure 4D). In contrast, W146 did not affect migration stimulated by MCP-1 (Figure 4D). Together, these data suggest that S1PR1 is required for HDL stimulated migration of macrophages.

### HDL stimulated macrophage migration is mediated by multiple downstream kinases

**Figure 5:**
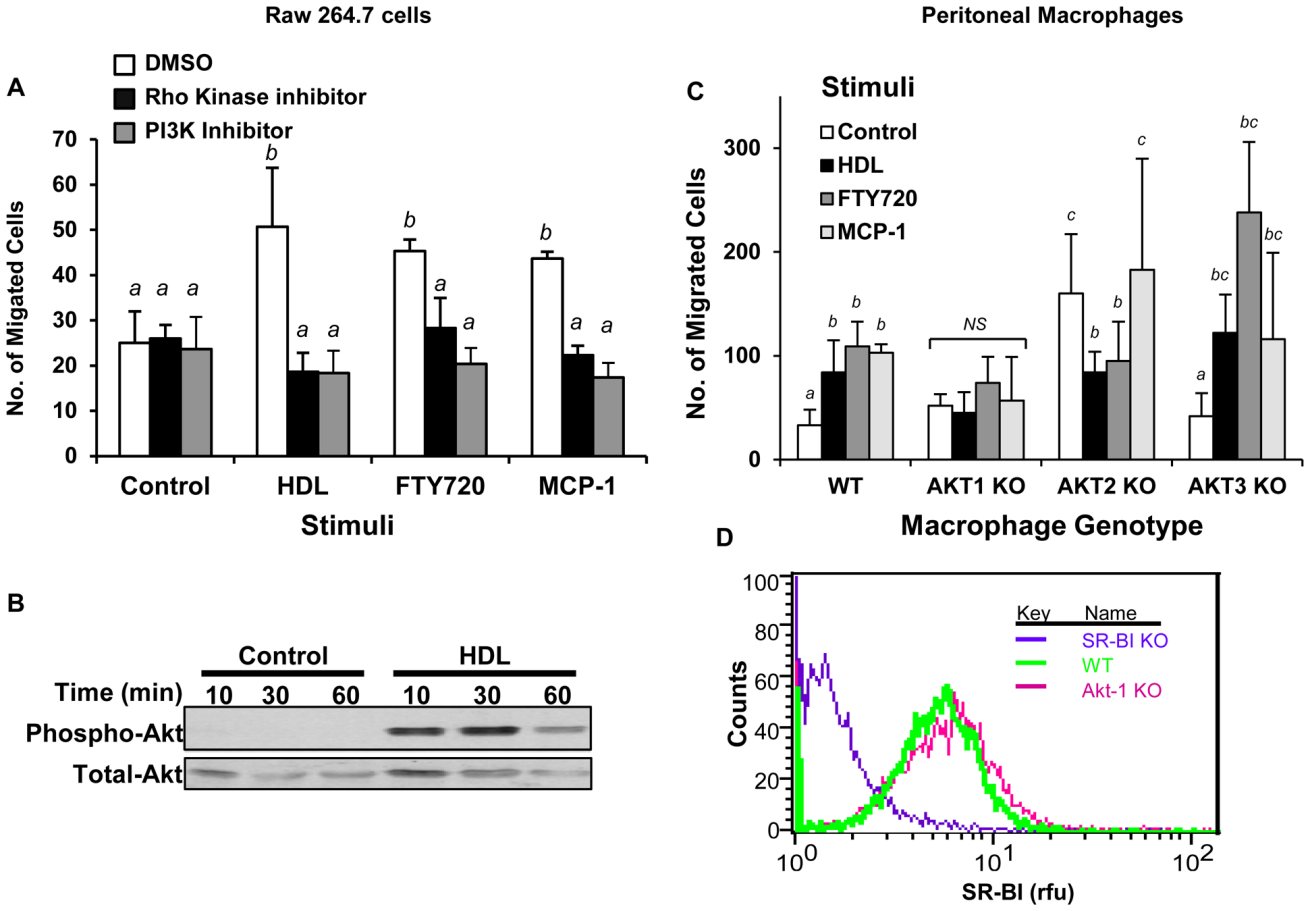
HDL stimulated macrophage migration involves Rho kinase and PI3K-Akt 1 signaling. **A.** RAW 264.7 cells were cultured in media containing 3% NCLPDS for 18 hrs. Cells were pre-incubated with 10 µM of either the Rho Kinase inhibitor, Y-27632, or the PI3K inhibitor LY294002, or with DMSO vehicle for 30 min and then the migration in response to HDL (100 µg protein/ml), FTY720 (2 ng/ml) or MCP-1 (100 ng/ml) was measured in the continued presence or absence of the indicated inhibitors. **B.** RAW 264.7 cells were serum starved for 18 hrs, washed and treated with or without HDL (100 µg protein/ml) for 10, 30 or 60 min. Equal amounts of proteins were analyzed by SDS-PAGE and immunoblotting for either phospho-Ser473- or total-Akt. **C.** MPM's were harvested from 6 independent WT, Akt1 KO, Akt2 KO or Akt3 KO mice. Migration in response to no stimulus, HDL (100 µg protein/ml), FTY720 (2 ng/ml) or MCP-1 (100 ng/ml) was measured. Data from A and C are means ± standard deviations of 6 replicates done over two independent experiments. Values identified with different letters are statistically significantly different (P<0.003, 2 way ANOVA with Tukey post hoc test). **D.** Flow cytometry analysis of cell surface SR-BI levels in wild type, SR-BI KO and Akt1 KO macrophages. Shown are representative histograms of an experiment performed twice.

Cell migration triggered by GPCR signaling has been reported to involve PI3K dependent pathways and PI3K independent pathways involving RhoA and Rho-kinase [92]-[97]. Consistent with this, pretreatment of RAW 264.7 cells with the Rho kinase inhibitor Y27632 or the PI3K inhibitor LY294002, did not affect basal migration, but reduced migration in response to HDL, FTY720 and MCP-1 (Figure 5A). Wortmannin, another PI3K inhibitor, had similar effects on HDL stimulated migration (data not shown). PI3K and protein kinase B/Akt have been implicated in driving initial steps of cell polarization and migrationin response to various stimuli [61], [98]-[102]. HDL (100 µg protein/ml) treatment of RAW 264.7 cells induced Akt phosphorylation as early as 10 min, with a peak at 30 min and diminished levels by 60 min (Figure 5B).

To determine which Akt isoform might mediate HDL stimulated migration in macrophages, we examined the ability of HDL to stimulate migration of peritoneal macrophages from KO mice lacking expression of either the Akt 1, 2 or 3 genes. Akt1 KO peritoneal macrophages exhibited slightly elevated basal migration compared to peritoneal macrophages from wild type mice (Figure 5C), but exhibited no increased migration in response to HDL, FTY720 or MCP-1. Akt2 KO peritoneal macrophages exhibited a five-fold increase in migration in the absence of stimulation compared to wild type cells. Migration of Akt2 KO macrophages was substantially reduced by HDL or FTY720, but was not affected by MCP-1, suggesting that Akt2 KO MPMs were still able to respond to both HDL and FTY720, albeit in the opposite manner as did wild type MPMs. On the other hand, HDL, FTY720 and MCP-1were able to stimulate increased migration of Akt3 KO macrophages (Figure 5C) suggesting that Akt3 was not required for migration in response to these stimuli. The inability of Akt1 KO MPMs to respond to HDL was not due to differences in SR-BI expression levels at the cell surface (Figure 5D). Similarly, Akt2 and Akt3 KO MPMs had normal cell surface levels of SR-BI (data not shown).

**Figure 6:**
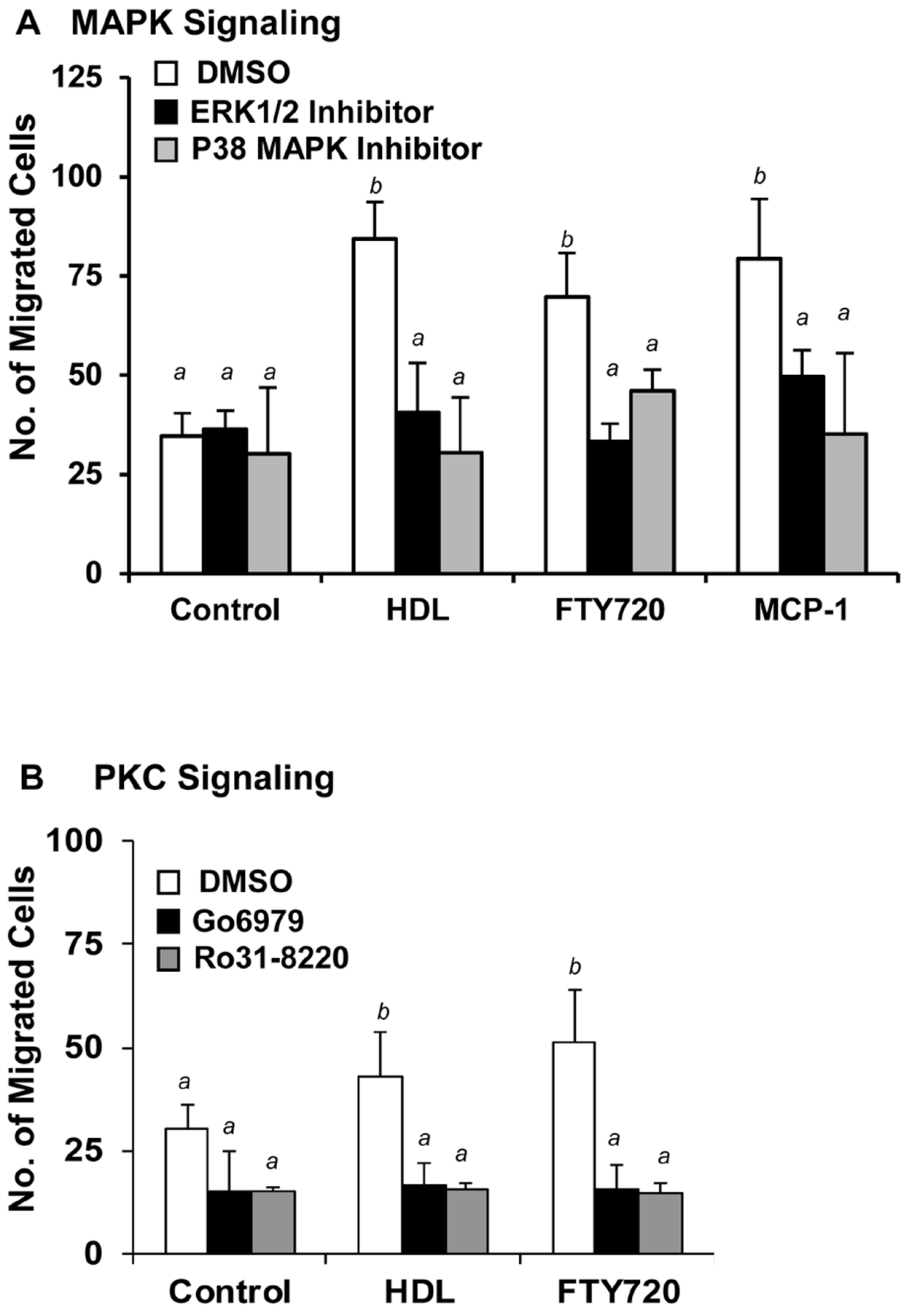
HDL stimulated macrophage migration involves MAP kinase and PKC pathways. RAW 264.7 cells were cultured in media containing 3%NCLPDS for 18 hrs. Cells were pre-incubated with the indicated inhibitors for 30 min and then the migration in response to HDL (100 µg protein/ml), FTY720 (2 ng/ml) or MCP-1 (100 ng/ml) was measured in the continued presence or absence of the indicated inhibitors. Control cells were treated with DMSO vehicle. **A.** Cells were treated with either 10 µM of the ERK1/2 pathway inhibitor, PD98059, or 1 µM of the p38 MAPK inhibitor, SB230580. **B.** Cells were treated with 5 µM of the PKC inhibitors Ro31-8220 or Go6976. Data are means ± standard deviations of 3 replicates. Values identified with different letters are statistically significantly different (P<0.04, 2 way ANOVA with Tukey post hoc test).

HDL induced activation of ERK1/2 and p38 MAPK has been reported in vascular smooth muscle cells, endothelial cells and in Chinese hamster ovary cells overexpressing SR-BI [37], [103], [104]. Therefore we tested the effects of inhibiting these kinase pathways on the ability of macrophages to migrate in response to HDL. Inhibition of the ERK1/2 pathway with PD98059, an inhibitor of ERK1/2 phosphorylation, did not affect basal macrophage migration but prevented macrophage migration in response to HDL, FTY720, and MCP-1 (Figure 6A). Similarly, inhibition of p38 MAPK activity with SB230580 also had no effect on basal macrophage migration but prevented migration in response to HDL, FTY720 and MCP-1 (Figure 6A). This is consistent with reports that both ERK1/2 and p38 MAPK pathways are involved in HDL and S1P induced migration of endothelial cells [61].

HDL also induces the activation of PKC in different cell types [37], [38], [105], and S1P induced cell migration has been shown to require PKC activity [106]. We therefore tested the involvement of PKC activity using a general PKC inhibitor, Ro31-8220, and an inhibitor of Ca^2+^-dependent PKC isoforms, Go6976 [107], [108]. Both of these inhibitors reduced basal migration and completely blocked the induction of migration of macrophages in response to HDL or FTY720 (Figure 6B), suggesting the involvement of a Ca^2+^-dependent PKC in HDL or FTY720 stimulated macrophage migration.

## Discussion

Macrophages are normally motile cells; however a number of studies have suggested that macrophages in atherosclerotic plaques exhibit impaired migration [5], [7]. Under some circumstances, however, migration can be re-established in macrophages in atherosclerotic plaques, and this appears to be important for atherosclerotic plaque regression, e.g. under conditions of elevated HDL levels [10], [11], [13]. The migration of MPM in response to native and modified lipoproteins has previously been demonstrated, however the underlying mechanisms were not investigated [109]. In the present study we show that HDL, but not lipid free apoA1 induced macrophage migration (Figure 1). This finding is consistent with studies that investigated the role of apoA1 in eNOS activation in endothelial cells, where it was shown that apoA1 is a necessary component of HDL but that lipid free apoA-I is not sufficient for stimulation of eNOS activation [110].

**Figure 7:**
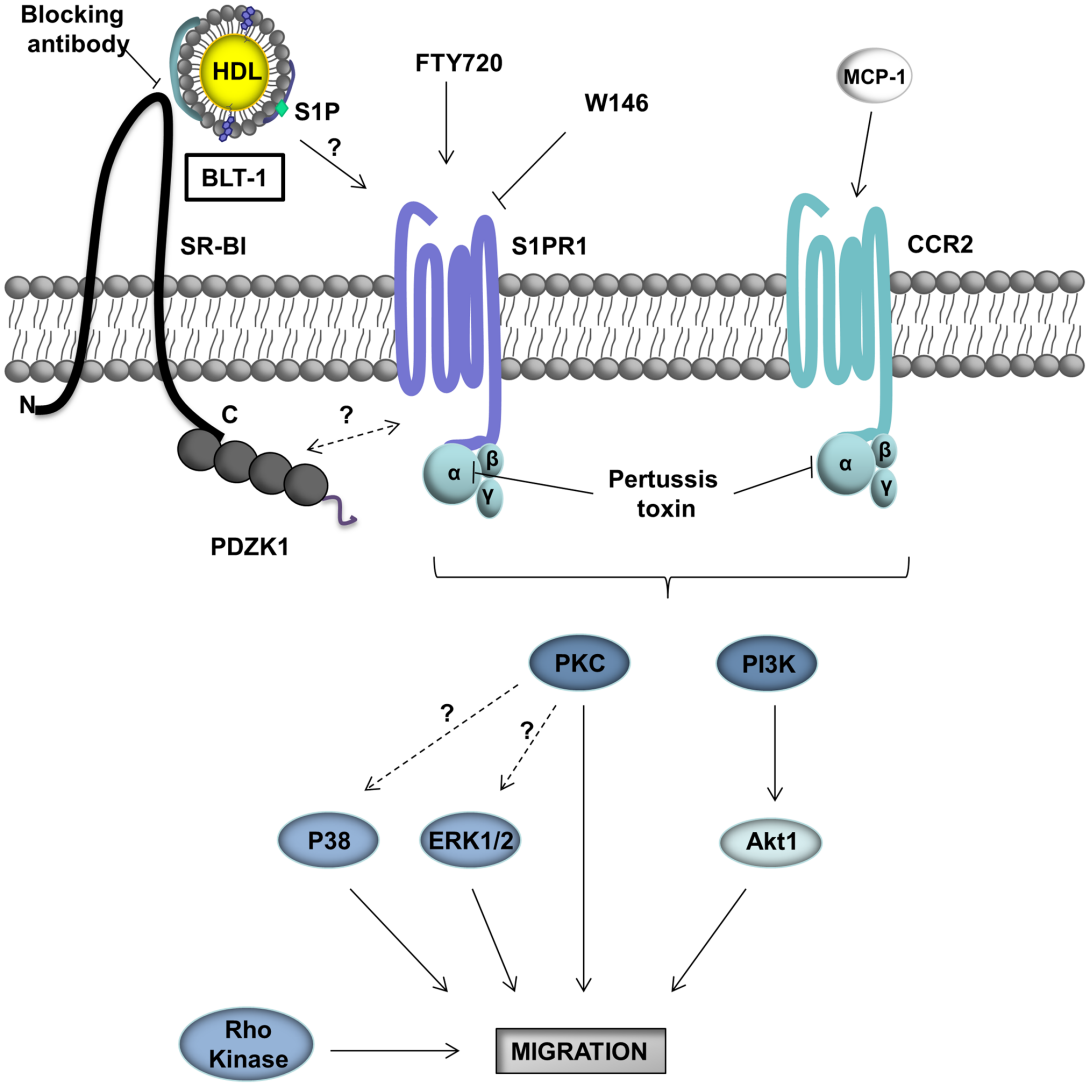
Working model for HDL mediated stimulation of macrophage migration. HDL binding to SR-BI leads to activation of S1PR1 signaling. This may involve transfer of S1P from bound HDL to S1PR1. Inhibition of HDL binding to SR-BI (blocking antibody) or SR-BI-mediated lipid transfer (BLT-1) prevents HDL dependent activation of S1PR1 signaling, but does not affect direct activation of S1PR1 by agonists. Inactivation of expression of SR-BI or PDZK1, on the other hand, inhibits migratory responses to FTY720, through an as yet unknown mechanism. HDL dependent activation of migration is suppressed by inhibition of S1PR1 signaling with FTY720 or W146, which directly antagonize S1PR1, or with PTX, which blocks Gα_i_ coupled GPCR's including CCR2 (receptor for MCP-1). Upon appropriate stimulation, S1PR1 (and CCR2) stimulate macrophage migration by activation of diverse signaling pathways including PI3K/Akt1, Rho kinase, PKC, p38 MAPK and Erk1/2 pathways .

Cell migration is a highly integrated, multistep process that involves dynamic rearrangement of the actin cytoskeleton and an extensive cross talk between signaling molecules at the leading edge of a migrating cell followed by a down regulation of adhesion molecules at the trailing end of the cell, reviewed in [92]. Lamellar extension is one of the early morphological changes in response to chemotactic stimuli [92]. We found that treatment of macrophages with HDL resulted in rapid (within 15 min) formation of lamellipodia in wild type macrophages but not those lacking SR-BI (Figure 2 B,C), consistent with the ability of HDL to stimulate chemotaxis of wild type macrophages but not macrophages in which SR-BI was inactivated or inhibited (Figure 2A, Figure 3), or macrophages that lacked the adaptor protein PDZK1 (Figure 2D) that binds to the C terminus of SR-BI. Our data also demonstrates that HDL induced the migration of macrophages in a manner that is sensitive to antagonists of the G-protein coupled receptor, S1PR1 (Figure 4A, B, D). The ability of an anti-SR-BI blocking antibody and an inhibitor of SR-BI meditated lipid transfer, BLT-1, to inhibit HDL but not FTY720 or MCP-1induced macrophage migration (Figure 3) demonstrate that HDL binding to SR-BI and SR-BI mediated lipid transfer activities are both required for macrophage migration in response to HDL, but not for migration in response to other chemotactic factors, including the S1P receptor pro-agonist FTY720 or MCP-1. This suggests that SR-BI may be involved in the uptake of HDL associated lipids such as sphingosine and/or S1P and may mediate their delivery to S1PRs, whereas the need for SR-BI activity can be bypassed in the absence of HDL by direct stimulation of S1PR1 with FTY720 (Figure 7). Alternatively, we cannot exclude the possibility that SR-BI mediated cholesterol efflux may also participate in HDL signaling to stimulate macrophage migration as has been suggested for endothelial cells (41) since BLT-1 also inhibits HDL dependent SR-BI mediated cholesterol efflux [111]. Further studies will be required to determine the mechanisms by which HDL binding to SR-BI and SR-BI mediated lipid transfer trigger signaling in macrophages.

Our data suggests that HDL-induced macrophage migration involves an S1PR, likely S1PR1. HDL is the major lipoprotein carrier of plasma S1P [53]. S1P is known to be a modulator of immune cell migration and appears to mediate the migration of endothelial cells treated with HDL [52], [58], [59]. Therefore we tested the involvement of S1PR activity in HDL dependent macrophage migration. The sphingosine analog FTY720, which is phosphorylated in vivo to FTY720-phosphate [112], a broad spectrum S1PR agonist, induced macrophage migration in a manner that was not diminished by the anti-SR-BI blocking antibody or by BLT-1 (Figure 4). This suggested that neither SR-BI's ability to bind HDL, nor its lipid transfer activity were required for macrophage migration in response to FTY720. Mouse macrophages were reported to express S1PR1 and S1PR2 and lower levels of S1PR3 [90]. S1PR2 negatively modulates macrophage migration in vivo and in vitro [91]. A number of lines of evidence implicate S1PR1 as the S1PR that mediates HDL dependent macrophage migration: First, HDL dependent macrophage migration is inhibited by PTX (Figure 3A), an inhibitor of Gα_i_ heterotrimeric G proteins, to which S1PR1 is coupled; second, an S1PR1 specific agonist, SEW2871, stimulates macrophage migration (Figure 3D); third, an S1PR1 specific antagonist,W146, prevents HDL stimulated macrophage migration (Figure 3D); fourth, we have detected S1PR1 but little to no S1PR3 expression in mouse peritoneal macrophages (Figure 4C). S1PR1 has previously been shown to be involved in HDL mediated eNOS activation, cell survival, migration and inhibition of adhesion molecule expression in endothelial cells [40], [61]. Our findings suggest that it also plays an important role in SR-BI dependent HDL stimulated migration of macrophages.

Surprisingly, macrophages from either SR-BI KO or PDZK1 KO mice were unable to migrate in response to the S1PR pro-agonist FTY720 (Figure 2A, D). This differed from the results of acutely inactivating SR-BI's HDL binding or lipid transfer activity (Figure 3), which impaired macrophage migration in response to HDL but left migration in response to FTY720 intact. The reasons why SR-BI KO or PDZK1 KO macrophages are unable to migrate in response to FTY720 is not clear and are the subject of on-going studies.

Our data suggests that HDL and the S1PR agonists FTY720 and SEW2871 share common downstream signaling pathways leading to macrophage migration which may suggests the involvement of common upstream signal transducers such as S1PR1. We demonstrate that HDL induced migration is dependent on the activation of PI3K-Akt1 signaling (Figure 5), consistent with the requirement of PI3K-Akt in HDL stimulated migration of endothelial cells [113]. PI3K-Akt signaling is known to be activated by S1PR1 [114]. The Akt family consists of three isoforms: Akt1, 2 and 3, encoded by different genes, all of which are reportedly expressed in macrophages [115]. Different Akt isoforms appear to play distinct roles in HDL stimulated macrophage migration. Interestingly, basal migration was enhanced ∼5 fold when Akt2 was knocked out (Figure 5C). This is consistent with previously reported observations that cell migration is enhanced in the absence of Akt2 expression and suggests that Akt2 negatively regulates basal migration [116]. Neither HDL nor FTY720 further increased migration of Akt2 KO macrophages, but rather, both reduced the enhanced basal migration by half (Figure 5C). This suggests that Akt2 KO MPMs could still respond to HDL or FTY720, although their response (reduced migration) was opposite that of wild type macrophages (increased migration) which contained Akt2. Akt3 did not play a significant role in migration in response to HDL, FTY720 or MCP-1 (Figure 5C). On the other hand, HDL induced migration was dependent on Akt1 (Figure 5C). Similar data were observed for FTY720 and MCP-1 stimulated migration. The difference in HDL mediated migration in Akt1, 2, and 3 KO macrophages was not due to differences in expression levels of SR-BI as these cells express similar levels of SR-BI compared to wild type macrophages (Figures 5D and data not shown). Akt isoforms seem to play non-redundant and sometimes opposing roles in cell migration [116] which appear to be cell type specific and depend on the chemotactic agent tested [117]–[119]. Our data suggests that Akt1 is downstream of HDL and is the key Akt isoform that mediates HDL induced migration of macrophages. Several studies reported the involvement of Akt1 in the development of atherosclerosis, macrophage inflammation, endothelial cell migration and vascular smooth muscle cell proliferation and protection against apoptosis [117], [120]–[123]. Overall these studies, together with our findings that Akt1 is required for HDL stimulated macrophage migration, are consistent with an atheroprotective function of Akt1. The requirement of Akt1 in HDL induced macrophage migration may suggest that at least some of the atheroprotective actions of HDL are mediated by Akt1.

In addition to the PI3K-Akt1 pathway, we used a chemical approach to demonstrate the involvement of p38 MAPK and ERK1/2 in HDL mediated macrophage migration (Figure 6 A). The involvement of p38 MAPK in HDL induced migration is similar to what has been reported previously for endothelial cells in which p38 MAPK was reported to act downstream of PI3K-Rac [61]. Unlike macrophages in which ERK1/2 appears to play a role in HDL stimulated migration, endothelial cell migration in response to HDL reportedly does not require ERK1/2 [61], suggesting cell type dependent differences in signaling pathways involved in HDL stimulated migration. We also demonstrate, using a chemical approach, the involvement of Ca^2+^-dependent PKCs in macrophage migration in response to HDL, FTY720 and MCP-1 (Figure 6B). HDL has been shown to increase intracellular calcium concentration in diverse cell types in culture, including, Chinese hamster ovary cells [124], human skin fibroblasts [125], [126], smooth muscle cells [127] and endothelial cells [54], [128]. Whether HDL also increases intracellular calcium in macrophages remains to be determined. Interestingly, Pilon et al. reported that SR-BI expression is increased in human adrenocortical cells in response to PKC activators, resulting in higher lipoprotein binding and specific cholesteryl ester uptake utilized for steroidogenesis [129], and we have previously reported that activation of PKC in transfected Chinese hamster ovary-derived and in HepG2 human hepatoma cells modulates the activity of SR-BI [37], [39]. Thus, SR-BI may be both a mediator and/or a target of HDL-dependent PKC activation.

Small GTPases of the Rho family play a key role in actin cytoskeleton organization and cell migration in response to chemokines and cytokines [92]–[96], [130], [131]. Blockage of Rho-kinase reduced HDL stimulated migration suggesting the requirement of this pathway in response to HDL (Figure 5A). Similar results were obtained for FTY720 and MCP-1 mediated migration consistent with the importance of the Rho-kinase pathway in macrophage migration in response to these stimuli. Although our data suggests the involvement of different kinases in HDL stimulated macrophage migration (Figure 7), the hierarchy and the interaction between multiple downstream signaling pathways remain to be established.

In conclusion we have demonstrated that HDL induces the migration of macrophages in an SR-BI, PDZK1 and S1PR1 dependent manner as summarized in Figure 7. HDL induced macrophage migration involves the PI3K-Akt1, p38MAPK, ERK1/2, PKC and Rho kinase pathways. HDL mediated macrophage migration may be one mechanism by which HDL contributes to protection against atherosclerosis development and atherosclerotic plaque regression.

## References

[1] Taylor PR, Martinez-Pomares L, Stacey M, Lin HH, Brown GD, et al (2005) Macrophage receptors and immune recognition. Annu Rev Immunol 23: 901–944. 15771589 10.1146/annurev.immunol.23.021704.115816

[2] Libby P (2002) Inflammation in atherosclerosis. Nature 420: 868–874. 12490960 10.1038/nature01323

[3] Steinberg D (1997) Low density lipoprotein oxidation and its pathobiological significance. J Biol Chem 272: 20963–20966. 9261091 10.1074/jbc.272.34.20963

[4] Tabas I, Williams KJ, Boren J (2007) Subendothelial lipoprotein retention as the initiating process in atherosclerosis: update and therapeutic implications. Circulation 116: 1832–1844. 17938300 10.1161/CIRCULATIONAHA.106.676890

[5] Nagao T, Qin C, Grosheva I, Maxfield FR, Pierini LM (2007) Elevated cholesterol levels in the plasma membranes of macrophages inhibit migration by disrupting RhoA regulation. Arterioscler Thromb Vasc Biol 27: 1596–1602. 17495238 10.1161/ATVBAHA.107.145086

[6] Park YM, Drazba JA, Vasanji A, Egelhoff T, Febbraio M, et al (2012) Oxidized LDL/CD36 interaction induces loss of cell polarity and inhibits macrophage locomotion. Mol Biol Cell 23: 3057–3068. 22718904 10.1091/mbc.E11-12-1051 PMC3418302

[7] Qin C, Nagao T, Grosheva I, Maxfield FR, Pierini LM (2006) Elevated plasma membrane cholesterol content alters macrophage signaling and function. Arterioscler Thromb Vasc Biol 26: 372–378. 16306428 10.1161/01.ATV.0000197848.67999.e1

[8] Ramkhelawon B, Yang Y, van Gils JM, Hewing B, Rayner KJ, et al (2013) Hypoxia induces netrin-1 and Unc5b in atherosclerotic plaques: mechanism for macrophage retention and survival. Arterioscler Thromb Vasc Biol 33: 1180–1188. 23599441 10.1161/ATVBAHA.112.301008 PMC3793633

[9] Feig JE, Pineda-Torra I, Sanson M, Bradley MN, Vengrenyuk Y, et al (2010) LXR promotes the maximal egress of monocyte-derived cells from mouse aortic plaques during atherosclerosis regression. J Clin Invest 120: 4415–4424. 21041949 10.1172/JCI38911 PMC2993578

[10] Feig JE, Rong JX, Shamir R, Sanson M, Vengrenyuk Y, et al (2011) HDL promotes rapid atherosclerosis regression in mice and alters inflammatory properties of plaque monocyte-derived cells. Proc Natl Acad Sci U S A 108: 7166–7171. 21482781 10.1073/pnas.1016086108 PMC3084076

[11] Llodra J, Angeli V, Liu J, Trogan E, Fisher EA, et al (2004) Emigration of monocyte-derived cells from atherosclerotic lesions characterizes regressive, but not progressive, plaques. Proc Natl Acad Sci U S A 101: 11779–11784. 15280540 10.1073/pnas.0403259101 PMC511052

[12] Randolph GJ (2008) Emigration of monocyte-derived cells to lymph nodes during resolution of inflammation and its failure in atherosclerosis. Curr Opin Lipidol 19: 462–468. 18769227 10.1097/MOL.0b013e32830d5f09 PMC2652166

[13] Trogan E, Feig JE, Dogan S, Rothblat GH, Angeli V, et al (2006) Gene expression changes in foam cells and the role of chemokine receptor CCR7 during atherosclerosis regression in ApoE-deficient mice. Proc Natl Acad Sci U S A 103: 3781–3786. 16537455 10.1073/pnas.0511043103 PMC1450154

[14] Williams KJ, Feig JE, Fisher EA (2008) Rapid regression of atherosclerosis: insights from the clinical and experimental literature. Nat Clin Pract Cardiovasc Med 5: 91–102. 18223541 10.1038/ncpcardio1086

[15] Feig JE, Quick JS, Fisher EA (2009) The role of a murine transplantation model of atherosclerosis regression in drug discovery. Curr Opin Investig Drugs 10: 232–238. PMC4662935 19333880

[16] Nissen SE, Tsunoda T, Tuzcu EM, Schoenhagen P, Cooper CJ, et al (2003) Effect of recombinant ApoA-I Milano on coronary atherosclerosis in patients with acute coronary syndromes: a randomized controlled trial. Jama 290: 2292–2300. 14600188 10.1001/jama.290.17.2292

[17] Raffai RL, Loeb SM, Weisgraber KH (2005) Apolipoprotein E promotes the regression of atherosclerosis independently of lowering plasma cholesterol levels. Arterioscler Thromb Vasc Biol 25: 436–441. 15591220 10.1161/01.ATV.0000152613.83243.12

[18] Reis ED, Li J, Fayad ZA, Rong JX, Hansoty D, et al (2001) Dramatic remodeling of advanced atherosclerotic plaques of the apolipoprotein E-deficient mouse in a novel transplantation model. J Vasc Surg 34: 541–547. 11533609 10.1067/mva.2001.115963

[19] Rong JX, Li J, Reis ED, Choudhury RP, Dansky HM, et al (2001) Elevating high-density lipoprotein cholesterol in apolipoprotein E-deficient mice remodels advanced atherosclerotic lesions by decreasing macrophage and increasing smooth muscle cell content. Circulation 104: 2447–2452. 11705823 10.1161/hc4501.098952

[20] Gordon DJ, Rifkind BM (1989) High-density lipoprotein—the clinical implications of recent studies. N Engl J Med 321: 1311–1316. 2677733 10.1056/NEJM198911093211907

[21] Assmann G, Schulte H (1992) Relation of high-density lipoprotein cholesterol and triglycerides to incidence of atherosclerotic coronary artery disease (the PROCAM experience). Prospective Cardiovascular Munster study. Am J Cardiol 70: 733–737. 1519522 10.1016/0002-9149(92)90550-i

[22] Assmann G, Schulte H, von Eckardstein A, Huang Y (1996) High-density lipoprotein cholesterol as a predictor of coronary heart disease risk. The PROCAM experience and pathophysiological implications for reverse cholesterol transport. Atherosclerosis 124 Suppl: S11–20 10.1016/0021-9150(96)05852-2 8831911

[23] Florentin M, Liberopoulos EN, Wierzbicki AS, Mikhailidis DP (2008) Multiple actions of high-density lipoprotein. Curr Opin Cardiol 23: 370–378. 18520722 10.1097/HCO.0b013e3283043806

[24] Nofer J, Kehrel B, Fobker M, Levkau B, Assmann G, von Eckardstein (2002) A (2002) HDL and arteriosclerosis: beyond reverse cholesterol transport. Atherosclerosis 161: 1–16. 11882312 10.1016/s0021-9150(01)00651-7

[25] Mineo C, Deguchi H, Griffin JH, Shaul PW (2006) Endothelial and antithrombotic actions of HDL. Circ Res 98: 1352–1364. 16763172 10.1161/01.RES.0000225982.01988.93

[26] Assmann G, Nofer JR (2003) Atheroprotective effects of high-density lipoproteins. Annu Rev Med 54: 321–341. 12414916 10.1146/annurev.med.54.101601.152409

[27] Hersberger M, von Eckardstein A (2005) Modulation of high-density lipoprotein cholesterol metabolism and reverse cholesterol transport. Handb Exp Pharmacol: 537–561. 10.1007/3-540-27661-0_20 16596814

[28] Cuchel M, Rader DJ (2006) Macrophage reverse cholesterol transport: key to the regression of atherosclerosis? Circulation 113: 2548–2555. 16735689 10.1161/CIRCULATIONAHA.104.475715

[29] Cho KH (2009) A reconstituted high density lipoprotein containing the V156E mutant of apolipoprotein A-I exhibits anti-atherosclerotic activity in Apo-E deficient mice. J Atheroscler Thromb 16: 217–229. 19638715 10.5551/jat.509

[30] Cho KH, Kim JR (2009) A reconstituted HDL containing V156K or R173C apoA-I exhibited anti-inflammatory activity in apo-E deficient mice and showed resistance to myeloperoxidase-mediated oxidation. Exp Mol Med 41: 417–428. 19322022 10.3858/emm.2009.41.6.047 PMC2705862

[31] Acton S, Rigotti A, Landschulz KT, Xu S, Hobbs HH, et al (1996) Identification of scavenger receptor SR-BI as a high density lipoprotein receptor. Science 271: 518–520. 8560269 10.1126/science.271.5248.518

[32] Covey SD, Krieger M, Wang W, Penman M, Trigatti BL (2003) Scavenger receptor class B type I-mediated protection against atherosclerosis in LDL receptor-negative mice involves its expression in bone marrow-derived cells. Arterioscler Thromb Vasc Biol 23: 1589–1594. 12829524 10.1161/01.ATV.0000083343.19940.A0

[33] Kozarsky KF, Donahee MH, Rigotti A, Iqbal SN, Edelman ER, et al (1997) Overexpression of the HDL receptor SR-BI alters plasma HDL and bile cholesterol levels. Nature 387: 414–417. 9163428 10.1038/387414a0

[34] Trigatti B, Rayburn H, Vinals M, Braun A, Miettinen H, et al (1999) Influence of the high density lipoprotein receptor SR-BI on reproductive and cardiovascular pathophysiology. Proc Natl Acad Sci U S A 96: 9322–9327. 10430941 10.1073/pnas.96.16.9322 PMC17781

[35] Ji Y, Jian B, Wang N, Sun Y, Moya ML, et al (1997) Scavenger receptor BI promotes high density lipoprotein-mediated cellular cholesterol efflux. J Biol Chem 272: 20982–20985. 9261096 10.1074/jbc.272.34.20982

[36] Norata GD, Catapano AL (2005) Molecular mechanisms responsible for the antiinflammatory and protective effect of HDL on the endothelium. Vasc Health Risk Manag 1: 119–129. 17315398 10.2147/vhrm.1.2.119.64083 PMC1993938

[37] Zhang Y, Ahmed AM, McFarlane N, Capone C, Boreham DR, et al (2007) Regulation of SR-BI-mediated selective lipid uptake in Chinese hamster ovary-derived cells by protein kinase signaling pathways. J Lipid Res 48: 405–416. 17079793 10.1194/jlr.M600326-JLR200

[38] Rentero C, Evans R, Wood P, Tebar F, Vila de Muga S, et al (2006) Inhibition of H-Ras and MAPK is compensated by PKC-dependent pathways in annexin A6 expressing cells. Cell Signal 18: 1006–1016. 16183252 10.1016/j.cellsig.2005.08.008

[39] Brunet R, How M, Trigatti BL (2011) Modulators of Protein Kinase C Affect SR-BI-Dependent HDL Lipid Uptake in Transfected HepG2 Cells. Cholesterol 2011: 687939. 21490774 10.1155/2011/687939 PMC3065880

[40] Kimura T, Tomura H, Mogi C, Kuwabara A, Damirin A, et al (2006) Role of scavenger receptor class B type I and sphingosine 1-phosphate receptors in high density lipoprotein-induced inhibition of adhesion molecule expression in endothelial cells. J Biol Chem 281: 37457–37467. 17046831 10.1074/jbc.M605823200

[41] Assanasen C, Mineo C, Seetharam D, Yuhanna IS, Marcel YL, et al (2005) Cholesterol binding, efflux, and a PDZ-interacting domain of scavenger receptor-BI mediate HDL-initiated signaling. J Clin Invest 115: 969–977. 15841181 10.1172/JCI200523858 PMC1069105

[42] Al-Jarallah A, Trigatti BL (2010) A role for the scavenger receptor, class B type I in high density lipoprotein dependent activation of cellular signaling pathways. Biochim Biophys Acta 1801: 1239–1248. 20732452 10.1016/j.bbalip.2010.08.006

[43] Mineo C, Shaul PW (2012) Functions of scavenger receptor class B, type I in atherosclerosis. Curr Opin Lipidol 23: 487–493. 22907331 10.1097/MOL.0b013e328357ba61

[44] Kocher O, Comella N, Tognazzi K, Brown LF (1998) Identification and partial characterization of PDZK1: a novel protein containing PDZ interaction domains. Lab Invest 78: 117–125. 9461128

[45] Silver DL (2002) A carboxyl-terminal PDZ-interacting domain of scavenger receptor B, type I is essential for cell surface expression in liver. J Biol Chem 277: 34042–34047. 12119305 10.1074/jbc.M206584200

[46] Yesilaltay A, Kocher O, Pal R, Leiva A, Quinones V, et al (2006) PDZK1 is required for maintaining hepatic scavenger receptor, class B, type I (SR-BI) steady state levels but not its surface localization or function. J Biol Chem 281: 28975–28980. 16867981 10.1074/jbc.M603802200

[47] Ikemoto M, Arai H, Feng D, Tanaka K, Aoki J, et al (2000) Identification of a PDZ-domain-containing protein that interacts with the scavenger receptor class B type I. Proc Natl Acad Sci U S A 97: 6538–6543. 10829064 10.1073/pnas.100114397 PMC18651

[48] Kocher O, Birrane G, Yesilaltay A, Shechter S, Pal R, et al (2011) Identification of the PDZ3 Domain of the Adaptor Protein PDZK1 as a Second, Physiologically Functional Binding Site for the C Terminus of the High Density Lipoprotein Receptor Scavenger Receptor Class B Type I. J Biol Chem 286: 25171–25186. 21602281 10.1074/jbc.M111.242362 PMC3137089

[49] Gong M, Wilson M, Kelly T, Su W, Dressman J, et al (2003) HDL-associated estradiol stimulates endothelial NO synthase and vasodilation in an SR-BI-dependent manner. J Clin Invest 111: 1579–1587. 12750408 10.1172/JCI16777 PMC155043

[50] Azhar S, Nomoto A, Reaven E (2002) Hormonal regulation of adrenal microvillar channel formation. J Lipid Res 43: 861–871. 12032160

[51] Okajima F, Sato K, Kimura T (2009) Anti-atherogenic actions of high-density lipoprotein through sphingosine 1-phosphate receptors and scavenger receptor class B type I. Endocr J 56: 317–334. 18753704 10.1507/endocrj.k08e-228

[52] Argraves KM, Argraves WS (2007) HDL serves as an S1P signaling platform mediating a multitude of cardiovascular effects. J Lipid Res. 10.1194/jlr.R700011-JLR200 17698855

[53] Murata N, Sato K, Kon J, Tomura H, Yanagita M, et al (2000) Interaction of sphingosine 1-phosphate with plasma components, including lipoproteins, regulates the lipid receptor-mediated actions. Biochem J 352 Pt 3: 809–815. PMC1221521 11104690

[54] Nofer JR, van der Giet M, Tolle M, Wolinska I, von Wnuck Lipinski K, et al (2004) HDL induces NO-dependent vasorelaxation via the lysophospholipid receptor S1P3. J Clin Invest 113: 569–581. 14966566 10.1172/JCI18004 PMC338256

[55] Sattler K, Levkau B (2009) Sphingosine-1-phosphate as a mediator of high-density lipoprotein effects in cardiovascular protection. Cardiovasc Res 82: 201–211. 19233866 10.1093/cvr/cvp070

[56] Frias MA, James RW, Gerber-Wicht C, Lang U (2009) Native and reconstituted HDL activate Stat3 in ventricular cardiomyocytes via ERK1/2: role of sphingosine-1-phosphate. Cardiovasc Res 82: 313–323. 19151362 10.1093/cvr/cvp024

[57] Matsuo Y, Miura S, Kawamura A, Uehara Y, Rye KA, et al (2007) Newly developed reconstituted high-density lipoprotein containing sphingosine-1-phosphate induces endothelial tube formation. Atherosclerosis 194: 159–168. 17118370 10.1016/j.atherosclerosis.2006.10.020

[58] Rivera J, Proia RL, Olivera A (2008) The alliance of sphingosine-1-phosphate and its receptors in immunity. Nat Rev Immunol 8: 753–763. 18787560 10.1038/nri2400 PMC2600775

[59] Takabe K, Paugh SW, Milstien S, Spiegel S (2008) "Inside-out" signaling of sphingosine-1-phosphate: therapeutic targets. Pharmacol Rev 60: 181–195. 18552276 10.1124/pr.107.07113 PMC2695666

[60] Hughes JE, Srinivasan S, Lynch KR, Proia RL, Ferdek P, et al (2008) Sphingosine-1-phosphate induces an antiinflammatory phenotype in macrophages. Circ Res 102: 950–958. 18323526 10.1161/CIRCRESAHA.107.170779 PMC2875063

[61] Kimura T, Sato K, Malchinkhuu E, Tomura H, Tamama K, et al (2003) High-density lipoprotein stimulates endothelial cell migration and survival through sphingosine 1-phosphate and its receptors. Arterioscler Thromb Vasc Biol 23: 1283–1288. 12775579 10.1161/01.ATV.0000079011.67194.5A

[62] Argraves KM, Gazzolo PJ, Groh EM, Wilkerson BA, Matsuura BS, et al (2008) High density lipoprotein-associated sphingosine 1-phosphate promotes endothelial barrier function. J Biol Chem 283: 25074–25081. 18606817 10.1074/jbc.M801214200 PMC2529014

[63] Liu F, Verin AD, Wang P, Day R, Wersto RP, et al (2001) Differential regulation of sphingosine-1-phosphate- and VEGF-induced endothelial cell chemotaxis. Involvement of G(ialpha2)-linked Rho kinase activity. Am J Respir Cell Mol Biol 24: 711–719. 11415936 10.1165/ajrcmb.24.6.4323

[64] Kimura T, Watanabe T, Sato K, Kon J, Tomura H, et al (2000) Sphingosine 1-phosphate stimulates proliferation and migration of human endothelial cells possibly through the lipid receptors, Edg-1 and Edg-3. Biochem J 348 Pt 1: 71–76. PMC1221037 10794715

[65] Morales-Ruiz M, Lee MJ, Zollner S, Gratton JP, Scotland R, et al (2001) Sphingosine 1-phosphate activates Akt, nitric oxide production, and chemotaxis through a Gi protein/phosphoinositide 3-kinase pathway in endothelial cells. J Biol Chem 276: 19672–19677. 11278592 10.1074/jbc.M009993200

[66] Rikitake Y, Hirata K, Kawashima S, Ozaki M, Takahashi T, et al (2002) Involvement of endothelial nitric oxide in sphingosine-1-phosphate-induced angiogenesis. Arterioscler Thromb Vasc Biol 22: 108–114. 11788469 10.1161/hq0102.101843

[67] Ryu Y, Takuwa N, Sugimoto N, Sakurada S, Usui S, et al (2002) Sphingosine-1-phosphate, a platelet-derived lysophospholipid mediator, negatively regulates cellular Rac activity and cell migration in vascular smooth muscle cells. Circ Res 90: 325–332. 11861422 10.1161/hh0302.104455

[68] Krieger M, Brown MS, Goldstein JL (1981) Isolation of Chinese hamster cell mutants defective in the receptor-mediated endocytosis of low density lipoprotein. J Mol Biol 150: 167–184. 6275086 10.1016/0022-2836(81)90447-2

[69] Theilmeier G, De Geest B, Van Veldhoven PP, Stengel D, Michiels C, et al (2000) HDL-associated PAF-AH reduces endothelial adhesiveness in apoE-/- mice. Faseb J 14: 2032–2039. 11023987 10.1096/fj.99-1029com

[70] Muller H, Hofer S, Kaneider N, Neuwirt H, Mosheimer B, et al (2005) The immunomodulator FTY720 interferes with effector functions of human monocyte-derived dendritic cells. Eur J Immunol 35: 533–545. 15657952 10.1002/eji.200425556

[71] Zhang X, Liu X, Shang H, Xu Y, Qian M (2011) Monocyte chemoattractant protein-1 induces endothelial cell apoptosis in vitro through a p53-dependent mitochondrial pathway. Acta Biochim Biophys Sin (Shanghai) 43: 787–795. 21859809 10.1093/abbs/gmr072

[72] Gu X, Kozarsky K, Krieger M (2000) Scavenger receptor class B, type I-mediated [3H]cholesterol efflux to high and low density lipoproteins is dependent on lipoprotein binding to the receptor. J Biol Chem 275: 29993–30001. 11001950 10.1074/jbc.275.39.29993

[73] Nieland TJ, Penman M, Dori L, Krieger M, Kirchhausen T (2002) Discovery of chemical inhibitors of the selective transfer of lipids mediated by the HDL receptor SR-BI. Proc Natl Acad Sci U S A 99: 15422–15427. 12438696 10.1073/pnas.222421399 PMC137732

[74] Murakami A, Takasugi H, Ohnuma S, Koide Y, Sakurai A, et al (2010) Sphingosine 1-phosphate (S1P) regulates vascular contraction via S1P3 receptor: investigation based on a new S1P3 receptor antagonist. Mol Pharmacol 77: 704–713. 20097776 10.1124/mol.109.061481

[75] Jajoo S, Mukherjea D, Pingle S, Sekino Y, Ramkumar V (2006) Induction of adenosine A1 receptor expression by pertussis toxin via an adenosine 5'-diphosphate ribosylation-independent pathway. J Pharmacol Exp Ther 317: 1–10. 16322354 10.1124/jpet.105.096255

[76] Chung EY, Psathas JN, Yu D, Li Y, Weiss MJ, et al (2012) CD19 is a major B cell receptor-independent activator of MYC-driven B-lymphomagenesis. J Clin Invest 122: 2257–2266. 22546857 10.1172/JCI45851 PMC3366393

[77] Andreadi CK, Howells LM, Atherfold PA, Manson MM (2006) Involvement of Nrf2, p38, B-Raf, and nuclear factor-kappaB, but not phosphatidylinositol 3-kinase, in induction of hemeoxygenase-1 by dietary polyphenols. Mol Pharmacol 69: 1033–1040. 16354769 10.1124/mol.105.018374

[78] Kim SK, Abdelmegeed MA, Novak RF (2006) The mitogen-activated protein kinase kinase (mek) inhibitor PD98059 elevates primary cultured rat hepatocyte glutathione levels independent of inhibiting mek. Drug Metab Dispos 34: 683–689. 16443668 10.1124/dmd.105.007666

[79] Verspohl EJ, Wienecke A (1998) The role of protein kinase C in the desensitization of rat pancreatic islets to cholinergic stimulation. J Endocrinol 159: 287–295. 9795370 10.1677/joe.0.1590287

[80] Mair KM, Robinson E, Kane KA, Pyne S, Brett RR, et al (2010) Interaction between anandamide and sphingosine-1-phosphate in mediating vasorelaxation in rat coronary artery. Br J Pharmacol 161: 176 192. 20718749 10.1111/j.1476-5381.2010.00878.x PMC2962826

[81] Sanna MG, Liao J, Jo E, Alfonso C, Ahn MY, et al (2004) Sphingosine 1-phosphate (S1P) receptor subtypes S1P1 and S1P3, respectively, regulate lymphocyte recirculation and heart rate. J Biol Chem 279: 13839–13848. 14732717 10.1074/jbc.M311743200

[82] Ayman S, Wallace P, Wayman CP, Gibson A, McFadzean I (2003) Receptor-independent activation of Rho-kinase-mediated calcium sensitisation in smooth muscle. Br J Pharmacol 139: 1532–1538. 12922941 10.1038/sj.bjp.0705394 PMC1573988

[83] Julien B, Grenard P, Teixeira-Clerc F, Van Nhieu JT, Li L, et al (2005) Antifibrogenic role of the cannabinoid receptor CB2 in the liver. Gastroenterology 128: 742–755. 15765409 10.1053/j.gastro.2004.12.050

[84] Li C, Yang G, Ruan J (2012) Sphingosine kinase-1/sphingosine-1-phosphate receptor type 1 signaling axis is induced by transforming growth factor-beta1 and stimulates cell migration in RAW264.7 macrophages. Biochem Biophys Res Commun 426: 415–420. 22960176 10.1016/j.bbrc.2012.08.108

[85] Bookout AL, Mangelsdorf DJ (2003) Quantitative real-time PCR protocol for analysis of nuclear receptor signaling pathways. Nucl Recept Signal 1: e012. 16604184 10.1621/nrs.01012 PMC1402222

[86] Kocher O, Yesilaltay A, Shen CH, Zhang S, Daniels K, et al (2008) Influence of PDZK1 on lipoprotein metabolism and atherosclerosis. Biochim Biophys Acta 1782: 310–316. 18342019 10.1016/j.bbadis.2008.02.004 PMC2421013

[87] Katada T (2012) The inhibitory G protein G(i) identified as pertussis toxin-catalyzed ADP-ribosylation. Biol Pharm Bull 35: 2103–2111. 23207763 10.1248/bpb.b212024

[88] Charo IF, Myers SJ, Herman A, Franci C, Connolly AJ, et al (1994) Molecular cloning and functional expression of two monocyte chemoattractant protein 1 receptors reveals alternative splicing of the carboxyl-terminal tails. Proc Natl Acad Sci U S A 91: 2752–2756. 8146186 10.1073/pnas.91.7.2752 PMC43448

[89] Davis MD, Clemens JJ, Macdonald TL, Lynch KR (2005) Sphingosine 1-phosphate analogs as receptor antagonists. J Biol Chem 280: 9833–9841. 15590668 10.1074/jbc.M412356200

[90] Keul P, Lucke S, von Wnuck Lipinski K, Bode C, Graler M, et al (2011) Sphingosine-1-phosphate receptor 3 promotes recruitment of monocyte/macrophages in inflammation and atherosclerosis. Circ Res 108: 314–323. 21164103 10.1161/CIRCRESAHA.110.235028

[91] Michaud J, Im DS, Hla T (2011) Inhibitory role of sphingosine 1-phosphate receptor 2 in macrophage recruitment during inflammation. J Immunol 184: 1475–1483. 10.4049/jimmunol.0901586 PMC3068864 20042570

[92] Jones GE (2000) Cellular signaling in macrophage migration and chemotaxis. J Leukoc Biol 68: 593–602. 11073096

[93] Jones GE, Ridley AJ, Zicha D (2000) Rho GTPases and cell migration: measurement of macrophage chemotaxis. Methods Enzymol 325: 449–462. 11036626 10.1016/s0076-6879(00)25465-7

[94] Ridley AJ (2004) Rho proteins and cancer. Breast Cancer Res Treat 84: 13–19. 14999150 10.1023/B:BREA.0000018423.47497.c6

[95] Ridley AJ, Allen WE, Peppelenbosch M, Jones GE (1999) Rho family proteins and cell migration. Biochem Soc Symp 65: 111–123. 10320936

[96] Ridley AJ (2001) Rho GTPases and cell migration. J Cell Sci 114: 2713–2722. 11683406 10.1242/jcs.114.15.2713

[97] Chodniewicz D, Zhelev DV (2003) Chemoattractant receptor-stimulated F-actin polymerization in the human neutrophil is signaled by 2 distinct pathways. Blood 101: 1181–1184. 12393389 10.1182/blood-2002-05-1435

[98] Burgering BM, Coffer PJ (1995) Protein kinase B (c-Akt) in phosphatidylinositol-3-OH kinase signal transduction. Nature 376: 599–602. 7637810 10.1038/376599a0

[99] Manning BD, Cantley LC (2007) AKT/PKB signaling: navigating downstream. Cell 129: 1261–1274. 17604717 10.1016/j.cell.2007.06.009 PMC2756685

[100] Sotsios Y, Ward SG (2000) Phosphoinositide 3-kinase: a key biochemical signal for cell migration in response to chemokines. Immunol Rev 177: 217–235. 11138779 10.1034/j.1600-065x.2000.17712.x

[101] Stephens L, Ellson C, Hawkins P (2002) Roles of PI3Ks in leukocyte chemotaxis and phagocytosis. Curr Opin Cell Biol 14: 203–213. 11891120 10.1016/s0955-0674(02)00311-3

[102] Vanhaesebroeck B, Leevers SJ, Ahmadi K, Timms J, Katso R, et al (2001) Synthesis and function of 3-phosphorylated inositol lipids. Annu Rev Biochem 70: 535–602. 11395417 10.1146/annurev.biochem.70.1.535

[103] Baranova IN, Vishnyakova TG, Bocharov AV, Kurlander R, Chen Z, et al (2005) Serum amyloid A binding to CLA-1 (CD36 and LIMPII analogous-1) mediates serum amyloid A protein-induced activation of ERK1/2 and p38 mitogen-activated protein kinases. J Biol Chem 280: 8031–8040. 15576377 10.1074/jbc.M405009200

[104] Mineo C, Yuhanna IS, Quon MJ, Shaul PW (2003) High density lipoprotein-induced endothelial nitric-oxide synthase activation is mediated by Akt and MAP kinases. J Biol Chem 278: 9142–9149. 12511559 10.1074/jbc.M211394200

[105] Mendez AJ, Oram JF, Bierman EL (1991) Protein kinase C as a mediator of high density lipoprotein receptor-dependent efflux of intracellular cholesterol. J Biol Chem 266: 10104–10111. 1645339

[106] Gorshkova I, He D, Berdyshev E, Usatuyk P, Burns M, et al (2008) Protein kinase C-epsilon regulates sphingosine 1-phosphate-mediated migration of human lung endothelial cells through activation of phospholipase D2, protein kinase C-zeta, and Rac1. J Biol Chem 283: 11794–11806. 18296444 10.1074/jbc.M800250200 PMC2431079

[107] Martiny-Baron G, Kazanietz MG, Mischak H, Blumberg PM, Kochs G, et al (1993) Selective inhibition of protein kinase C isozymes by the indolocarbazole Go 6976. J Biol Chem 268: 9194–9197. 8486620

[108] Nixon JS, Bishop J, Bradshaw D, Davis PD, Hill CH, et al (1992) The design and biological properties of potent and selective inhibitors of protein kinase C. Biochem Soc Trans 20: 419–425. 1397642 10.1042/bst0200419

[109] Trach CC, Wulfroth PM, Severs NJ, Robenek H (1996) Influence of native and modified lipoproteins on migration of mouse peritoneal macrophages and the effect of the antioxidants vitamin E and Probucol. Eur J Cell Biol 71: 199–205. 8905298

[110] Yuhanna IS, Zhu Y, Cox BE, Hahner LD, Osborne-Lawrence S, et al (2001) High-density lipoprotein binding to scavenger receptor-BI activates endothelial nitric oxide synthase. Nat Med 7: 853–857. 11433352 10.1038/89986

[111] Nieland TJ, Chroni A, Fitzgerald ML, Maliga Z, Zannis VI, et al (2004) Cross-inhibition of SR-BI- and ABCA1-mediated cholesterol transport by the small molecules BLT-4 and glyburide. J Lipid Res 45: 1256–1265. 15102890 10.1194/jlr.M300358-JLR200

[112] Billich A, Bornancin F, Devay P, Mechtcheriakova D, Urtz N, et al (2003) Phosphorylation of the immunomodulatory drug FTY720 by sphingosine kinases. J Biol Chem 278: 47408–47415. 13129923 10.1074/jbc.M307687200

[113] Seetharam D, Mineo C, Gormley AK, Gibson LL, Vongpatanasin W, et al (2006) High-density lipoprotein promotes endothelial cell migration and reendothelialization via scavenger receptor-B type I. Circ Res 98: 63–72. 16339487 10.1161/01.RES.0000199272.59432.5b

[114] Guo H, Zhao Z, Yang Q, Wang M, Bell RD, et al (2013) An activated protein C analog stimulates neuronal production by human neural progenitor cells via a PAR1-PAR3-S1PR1-Akt pathway. J Neurosci 33: 6181–6190. 23554499 10.1523/JNEUROSCI.4491-12.2013 PMC3707621

[115] Shiratsuchi H, Basson MD (2007) Akt2, but not Akt1 or Akt3 mediates pressure-stimulated serum-opsonized latex bead phagocytosis through activating mTOR and p70 S6 kinase. J Cell Biochem 102: 353–367. 17372934 10.1002/jcb.21295

[116] Zhou GL, Tucker DF, Bae SS, Bhatheja K, Birnbaum MJ, et al (2006) Opposing roles for Akt1 and Akt2 in Rac/Pak signaling and cell migration. J Biol Chem 281: 36443–36453. 17012749 10.1074/jbc.M600788200

[117] Arboleda MJ, Lyons JF, Kabbinavar FF, Bray MR, Snow BE, et al (2003) Overexpression of AKT2/protein kinase Bbeta leads to up-regulation of beta1 integrins, increased invasion, and metastasis of human breast and ovarian cancer cells. Cancer Res 63: 196–206. 12517798

[118] Irie HY, Pearline RV, Grueneberg D, Hsia M, Ravichandran P, et al (2005) Distinct roles of Akt1 and Akt2 in regulating cell migration and epithelial-mesenchymal transition. J Cell Biol 171: 1023–1034. 16365168 10.1083/jcb.200505087 PMC2171329

[119] Yoeli-Lerner M, Yiu GK, Rabinovitz I, Erhardt P, Jauliac S, et al (2005) Akt blocks breast cancer cell motility and invasion through the transcription factor NFAT. Mol Cell 20: 539–550. 16307918 10.1016/j.molcel.2005.10.033

[120] Ackah E, Yu J, Zoellner S, Iwakiri Y, Skurk C, et al (2005) Akt1/protein kinase Balpha is critical for ischemic and VEGF-mediated angiogenesis. J Clin Invest 115: 2119–2127. 16075056 10.1172/JCI24726 PMC1180542

[121] Androulidaki A, Iliopoulos D, Arranz A, Doxaki C, Schworer S, et al (2009) The kinase Akt1 controls macrophage response to lipopolysaccharide by regulating microRNAs. Immunity 31: 220–231. 19699171 10.1016/j.immuni.2009.06.024 PMC2865583

[122] Fernandez-Hernando C, Ackah E, Yu J, Suarez Y, Murata T, et al (2007) Loss of Akt1 leads to severe atherosclerosis and occlusive coronary artery disease. Cell Metab 6: 446–457. 18054314 10.1016/j.cmet.2007.10.007 PMC3621848

[123] Fernandez-Hernando C, Jozsef L, Jenkins D, Di Lorenzo A, Sessa WC (2009) Absence of Akt1 reduces vascular smooth muscle cell migration and survival and induces features of plaque vulnerability and cardiac dysfunction during atherosclerosis. Arterioscler Thromb Vasc Biol 29: 2033–2040. 19762778 10.1161/ATVBAHA.109.196394 PMC2796372

[124] Grewal T, Evans R, Rentero C, Tebar F, Cubells L, et al (2005) Annexin A6 stimulates the membrane recruitment of p120GAP to modulate Ras and Raf-1 activity. Oncogene 24: 5809–5820. 15940262 10.1038/sj.onc.1208743

[125] Nofer JR, Fobker M, Hobbel G, Voss R, Wolinska I, et al (2000) Activation of phosphatidylinositol-specific phospholipase C by HDL-associated lysosphingolipid. Involvement in mitogenesis but not in cholesterol efflux. Biochemistry 39: 15199–15207. 11106499 10.1021/bi001162a

[126] Porn MI, Akerman KE, Slotte JP (1991) High-density lipoproteins induce a rapid and transient release of Ca2+ in cultured fibroblasts. Biochem J 279 (Pt 1): 29–33. 10.1042/bj2790029 PMC1151542 1930148

[127] Bochkov V, Tkachuk V, Buhler F, Resink T (1992) Phosphoinositide and calcium signaling responses in smooth muscle cells: comparison between lipoproteins, Ang II, and PDGF. Biochem Biophys Res Commun 188: 1295–1304. 1332716 10.1016/0006-291x(92)91372-w

[128] Honda HM, Wakamatsu BK, Goldhaber JI, Berliner JA, Navab M, et al (1999) High-density lipoprotein increases intracellular calcium levels by releasing calcium from internal stores in human endothelial cells. Atherosclerosis 143: 299–306. 10217358 10.1016/s0021-9150(98)00302-5

[129] Pilon A, Martin G, Bultel-Brienne S, Junquero D, Delhon A, et al (2003) Regulation of the scavenger receptor BI and the LDL receptor by activators of aldosterone production, angiotensin II and PMA, in the human NCI-H295R adrenocortical cell line. Biochim Biophys Acta 1631: 218–228. 12668173 10.1016/s1388-1981(03)00020-9

[130] Ridley AJ (2001) Rho family proteins: coordinating cell responses. Trends Cell Biol 11: 471–477. 11719051 10.1016/s0962-8924(01)02153-5

[131] Ridley AJ (2001) Rho proteins: linking signaling with membrane trafficking. Traffic 2: 303–310. 11350626 10.1034/j.1600-0854.2001.002005303.x

