# Correction: High Density Lipoprotein Stimulated Migration of Macrophages Depends on the Scavenger Receptor Class B, Type I, PDZK1 and Akt1 and Is Blocked by Sphingosine 1 Phosphate Receptor Antagonists

**DOI:** 10.1371/journal.pone.0117623

**Published:** 2015-01-09

**Authors:** The PLOS ONE Staff

The current address for the first author is not indicated. The current address for Aishah Al-Jarallah is Department of Biochemistry, Faculty of Medicine, Kuwait University, P.O. Box 24 923, 13 110 Safat, Kuwait.
